# A dose-finding design for phase I clinical trials based on Bayesian stochastic approximation

**DOI:** 10.1186/s12874-022-01741-3

**Published:** 2022-10-01

**Authors:** Jin Xu, Dapeng Zhang, Rongji Mu

**Affiliations:** 1 School of Statistics, East China Normal University, 3663 North Zhongshan Road, 200062 Shanghai, China; 2 Key Laboratory of Advanced Theory and Application in Statistics and Data Science - MOE, East China Normal University, Shanghai, China; 3 Clinical Research Center, Shanghai Jiao Tong University School of Medicine, Shanghai, China

**Keywords:** Adaptive design, Dose-finding, Maximum tolerated dose, Stochastic approximation

## Abstract

**Background:**

Current dose-finding designs for phase I clinical trials can correctly select the MTD in a range of 30–80% depending on various conditions based on a sample of 30 subjects. However, there is still an unmet need for efficiency and cost saving.

**Methods:**

We propose a novel dose-finding design based on Bayesian stochastic approximation. The design features utilization of dose level information through local adaptive modelling and free assumption of toxicity probabilities and hyper-parameters. It allows a flexible target toxicity rate and varying cohort size. And we extend it to accommodate historical information via prior effective sample size. We compare the proposed design to some commonly used methods in terms of accuracy and safety by simulation.

**Results:**

On average, our design can improve the percentage of correct selection to about 60% when the MTD resides at a early or middle position in the search domain and perform comparably to other competitive methods otherwise. A free online software package is provided to facilitate the application, where a simple decision tree for the design can be pre-printed beforehand.

**Conclusion:**

The paper proposes a novel dose-finding design for phase I clinical trials. Applying the design to future cancer trials can greatly improve the efficiency, consequently save cost and shorten the development period.

**Supplementary Information:**

The online version contains supplementary material available at 10.1186/s12874-022-01741-3.

## Introduction

The past decade has witnessed a fast growing trend in investigational new drug application and early phase clinical trial. However, the probability of (transition) success for phase I trials is still as low as about 50%, especially for oncology drugs [1]. The quality and performance of dose-finding design becomes the key to the success for late phase development. We focus on the design to determine the maximum tolerated dose (MTD) for anti-cancer drugs where the probability of toxicity is assumed monotonically increasing in dose. Given a finite sample as small as 30 subjects, the current designs can correctly select/estimate the MTD at about 50% [2], which still has substantial room for improvement to meet clinical needs. Denote the probability of experiencing dose-limiting toxicity (DLT) event at dose *x* by $\pi (x)=\mathrm {Pr}(y=1|x)$, where *y* is the binary response of DLT. Then, in a nutshell, given a prespecified acceptable toxicity rate $\alpha$, such as 20% or 30%, the dose-finding design can be treated as a sequential estimation of the quantile from a finite collection of candidate doses $\mathcal {C}$, i.e., $d^*=\mathrm {argmin}_{d\in \mathcal {C}}|\pi (d)-\alpha |$.

There is a rich literature on methods for this problem, which can generally be classified in three categories: the algorithm-based methods, the model-based methods, and the model-assisted methods. The $3+3$ method [3] is the most popular algorithm-based design. Simple rules for dose-escalation make it easy to use and accessible by clinicians. Even though it is inefficient with a correct selection rate as low as 30% [4, 5], it is still widely adopted by pharmaceutical companies and authorities. The model-based methods allow borrowing information across doses. They usually consist of two steps. The first step is to postulate some parametric model for the underlying dose response curve. The second step uses a Bayesian paradigm to update the posterior distribution of the parameters and the induced distribution of the toxicity rate upon which the decision for the next dose is made. Some popular methods include the continual reassessment method (CRM) [6], the escalation with overdose control (EWOC) [7, 8] and the Bayesian logistic regression model (BLRM) [9]. Recently, model-assisted interval designs have gained popularity. Such designs first partition the toxicity probability space into three subintervals representing decision regions of escalation, stay and de-escalation, respectively. The designs decide the next dose based on the posterior coverage of the three intervals. Specifically, Ji et al. [10] proposed a modified toxicity probability interval (mTPI) to sequentially determine the next dose based on unit probability mass. An improved version, called mTPI-2, was proposed by Guo et al. [11] to avoid some suboptimal solution. It was later showed to be equivalent to the Keyboard design of Yan et al. [2, 12]. Liu and Yuan [13] proposed a Bayesian optimal interval (BOIN) design which determines the next dose by thresholding the observed toxicity rate to minimize the decision error from a hypothesis testing perspective.

One important advantage of the rule-based methods, including both algorithm-based and model-assisted methods, is transparency. A statistician can tabulate all scenarios of possible outcomes and the corresponding decisions for dose assignment in advance so that clinicians can implement it easily without resorting to extra computational tools. On the other hand, the inefficiency of these rule-based methods stems from the ignorance of the exact dose information (as only ranks of doses are used) and the trajectory of the search path. The 3+3 method targets the DLT probability from 1/6 to 1/3 and only uses the frequency information at the current dose to make decisions [14]. The model-based methods can lose efficiency in two aspects. First, they may overly emphasize the *global* fit for the dose-toxicity curve while the actual interest is the point estimation of a *local* quantile. For example, it is not wise to use data collected at low dose levels to update the distribution of parameters while the MTD resides in the high dose region. Second, the methods rely on some subjective assumptions/specifications about priors, hyper-parameters, and threshold values. Different specifications can lead to significantly different results in MTD selection.

In a different route, the quantile estimation problem has been dealt with using stochastic approximation [15] in sensitivity experiments. Some useful variants have been proposed to improve efficiency over the years [16, 17]. However, for the dose-finding problem, it suffers from the discrete barrier [18].

In this paper, we attempt to fix these inefficiency problems and improve the estimation accuracy. We propose a novel design based on the Bayesian stochastic approximation method introduced by Xu et al. [19] The idea is to employ a local linear model and a Bayesian scheme to sequentially estimate the interested quantile directly alone the search path. We show that the design has the desired properties of coherence and consistency. Moreover, it enjoys simplicity in the sense that neither pre-specification of toxicity probabilities nor subjective priors for parameters are required. We show the design can be extended to incorporate historical information through prior effective sample size. Extensive simulation demonstrates its superior performance to some popular methods in terms of accuracy and overdose control. On average, based on a sample of 30 subjects the proposed design can correctly select the MTD at about 60% when the MTD resides at a early or middle position in the search domain and perform comparably to other competitive methods otherwise.

A free package at https://bsa4df.shinyapp.io/BSA_app is provided to facilitate the application with a pre-printed decision tree for the next three cohorts that can to be easily used by practitioners.

## Methods

### Design

Suppose *K* doses are considered. To facilitate the model, we need two simple preprocesses. The first preprocess is to scale the dose levels in (0,1] and sort them in ascending order in $\mathcal {C} = \{d_1,\ldots ,d_K\}$. This can be done by a linear map based on the range of the dose levels in their original scale or log scale so that the dose levels are well spread over the interval (0, 1]. For example, the dose levels 1, 2, 3, 4, 5 are converted to 0.1, 0.3, 0.5, 0.7, 0.9 by the transformation $(x-0.5)/5$ and the dose levels 100, 200, 400, 800 are converted to 0.28, 0.59, 0.77, 0.91 by the transformation $\{\log (x)-4\}/2.2$(A generic rule is described in Supplementary Materials.). The second preprocess is to divide (0, 1] equally into *s* subintervals. It is essential for local modeling as we shall focus on one of these subintervals at a time. The value *s* decides the length of subintervals. Due to the scarcity of sample size in phase I trial, we recommend a small integer such as 3 or 4 in practice. Sensitivity analysis is provided later.

We apply the Bayesian stochastic approximation (BSA) [19] to construct the sequential design. The procedure is summarized as follows.

Fix the cohort size to be three as usual. We start to treat the first cohort at the lowest dose level. For $n=1,2,\ldots$, suppose the dose level for the current *n*th cohort is $x_n$, which corresponds to $d_k$. Then, $x_n$ is uniquely contained in the subinterval $(v_0,v_1]$, where $v_0=(\lceil x_ns \rceil -1)/s$, $v_1=\lceil x_ns \rceil /s$ and $\lceil \cdot \rceil$ is the ceiling function. For example, $x_n=0.2\in (0,1/3]$ with $s=3$. Let $y_{na}$ denote the binary response of the *a*th patient of cohort *n*. Denote the cumulative data up to the *n*th cohort by $\mathcal {D}_n=\{(x_i,y_{ia}): i=1,\ldots ,n, a=1,\ldots ,3\}$. It is worth noting that the design allows varying cohort sizes throughout the trial, e.g., three subjects for cohort one and one subject for cohort two. We provide more discussion in a sensitivity analysis.

Let $\theta$ denote the root of $\pi (x)=\alpha$, i.e., $\theta =\pi ^{-1}(\alpha )$. It is unique when we assume $\pi (x)$ is strictly increasing. First, approximate $\pi (x)$ in $(v_0,v_1]$ by a segment of the line through the point $(\theta ,\alpha )$ given by $F(x) = \alpha + \beta (x-\theta )$. See the illustration in Fig. S1. Denote the values of the line segment at the two ends by $\rho _0=F(v_0)$ and $\rho _1=F(v_1)$. By the monotonicity of $\pi$, assume $\beta$ is positive. And, we have $\rho _0<\rho _1$. The local adaptive modeling focuses on the neighborhood of the current dose and minimizes the unnecessary influence of ‘outsider’ data. It improves the efficiency of the subsequent Bayesian estimation. The linear model suffices for the local modeling and enjoys the simplicity for computation and interpretation.

Second, we impose a noninformative uniform prior for $(\rho _0,\rho _1)$ (with the density $2I(0<\rho _0<\rho _1<1)$, where *I* is the indicator function). This prior reflects the monotonicity assumption of the dose-toxicity relationship precisely. It avoids subjective specification of the prior of variables that are parameterized in some other way, e.g., intercept and slope as in Babb et al. [7] and Neuenschwander et al. [9]. Based on this prior, we derive the induced joint prior for $(\beta ,\theta )$.

Third, using the data with doses contained in the current subinterval $(v_0,v_1]$, compute the posterior distribution of $(\beta ,\theta )$. Then, compute the marginal posterior of $\theta$, denoted by $h(\theta )$, and the posterior mean of $\theta$, denoted by $\mathrm {E}(\theta |\mathcal {D}_n)$, the latter of which is the Bayes estimator of the desired quantile. One remarkable consequence of the noninformative prior is that the posterior distribution of $\theta$ can be obtained recursively and analytically so that the computation is simple without resorting to Markov chain Monte Carlo. (See details in Xu et al. [19])

At last, determine the dose for the next cohort to be the dose in the neighborhood of the current dose which is closest to the posterior mean of the target dose, i.e., $x_{n+1} =d_{a^*}$, where

1 $$\begin{aligned} a^* = \mathrm {argmin}\left\{ |d_a - \mathrm {E}(\theta |\mathcal {D}_n)|: a=\max (k-1,1),k,\min (k+1,K)\right\} . \end{aligned}$$

In this way, dose skipping is not allowed as usually required in practice.

The proposed design possesses two desirable properties. First, the design satisfies the coherence property introduced by Cheung [20] in the sense that the probability of dose escalation (or de-escalation) is 0 when the DLT response is 1 (or 0) at the current dose (See the proof in Supplementary Materials.). This finite sample property is appealing to clinicians in that dose escalation is not allowed when a DLT event occurs. Second, by Proposition 2 of Xu et al. [19] and Theorem 1 of Oron et al. [21] we have the consistency of the estimator, i.e., $x_n$ converges to $d^*$ almost surely.

### Algorithm

We summarize the algorithm for the proposed design as follows.

Step 1: Treat the first cohort at the lowest dose $d_1$.
Step 2: At current dose $d_k$, determine $(v_0,v_1]$ and the data contained in this subinterval. Compute the posterior distribution of $\theta$ and determine the next dose by (1).
Step 3: Repeat Step 2 until the sample size is exhausted. Choose the next dose as MTD.

In practice, a couple of quick actions can be taken to expedite the algorithm without invoking Bayesian calculation in Step 2. First, escalate before having the very first occurrence of DLT. That is if no DLT event has ever occurred up to the current dose $d_k$, conduct the experiment for the next cohort at dose $d_{k+1}$ whenever it is defined. Such step is adopted by Simon et al. [22] and Riviere et al. [23].

Second, in Step 2, after accumulating a certain number, say $m_0$, of patients at one dose, we construct a Wald-type interval to make quick decision for the next dose with high confidence. To be specific, let $m_k$ denote the number of patients treated at $d_k$ and let $\widehat{p}_k$ denote the relative frequency of DLT at $d_k$. (We apply the pool-adjacent-violators algorithm to keep the monotonicity of $\widehat{p}_k$. I.e., replace $\widehat{p}_k$ with the average of the relative frequency of DLT at $d_k$ and the relative frequency of DLT at the previous dose if they are not monotonic [13].) Let $\mathrm {logit}(x)=\log \{x/(1-x)\}$ denote the logit function. When $m_k$ is large, say no less than $m_0$ in our case, by large sample theory and delta method, $\mathrm {logit}(\widehat{p}_k)$ is approximately distributed as $N(\mathrm {logit}(p_k),\{m_kp_k(1-p_k)\}^{-1})$. Denote $z_\xi$ as the upper $100\xi$ percentile of the standard normal distribution. If $p_k$ is close to $\alpha$, then with probability of $1-2\xi$, $\mathrm {logit}(\widehat{p}_k)$ falls in the interval $(c_1,c_2)=(\mathrm {logit}(\alpha ) - z_\xi \{m_k \alpha (1-\alpha )\}^{-1/2}, \mathrm {logit}(\alpha ) + z_\xi \{m_k \alpha (1-\alpha )\}^{-1/2})$. Or equivalently $\widehat{p}_k$ falls in the interval $(\{1+\exp {(-c_1)}\}^{-1}, \{1+\exp {(-c_2)}^{-1}\})$. (Note that the logit transformation makes the distribution of $\mathrm {logit}(\widehat{p}_k)$ more symmetric (or less skewed) than $\widehat{p}_k$.) If $\widehat{p}_k$ is smaller than the lower limit of the interval indicating $d_k$ is below the MTD with high confidence, escalate to $\min (d_{k+1},d_K)$. If $\widehat{p}_k$ is greater than the upper limit of the interval indicating that $d_k$ is above the MTD with high confidence, de-escalate to $\max (d_{k-1},d_1)$. In particular, if $\widehat{p}_1$ is greater than the upper bound of the interval, we shall terminate the trial for toxicity [11]. Otherwise, when $\widehat{p}_k$ falls in the interval, the proposed Bayesian method is used to make the transition decision. To have high confidence, we set $\xi =0.05$ that corresponds to a 90% confidence interval and $m_0=12$ [13]. In fact, a large number of experiments, such as 12 out of 30, on a particular dose indicates strongly the convergence of MTD.

### Example

We illustrate the application of the proposed design and algorithm by an example used in O’Quigley et al. [6]. The dose-toxicity response curve is generated by $\mathrm {Pr}(y=1)=[\{\mathrm {tanh}(x)+1\}/2]^2$, where six candidate levels are chosen as follows.

$$\begin{aligned} \begin{array}{c c cccccc} k &{}&{} 1 &{} 2 &{} 3 &{} 4 &{} 5 &{} 6\\ \text {original dose level} &{}\quad &{} -1.47 &{} -1.1&{} -0.69&{} -0.42&{} 0.0 &{} 0.42\\ \text {scaled dose level} &{}\quad &{} 0.015 &{} 0.20 &{} 0.405 &{} 0.54 &{} 0.75 &{} 0.96 \\ \text {toxicity probability} &{}\quad &{} 0.002 &{} 0.01 &{} 0.04 &{} 0.09 &{} 0.24 &{} 0.49\\ \end{array} \end{aligned}$$

The target toxicity rate is $20\%$. Thus, the MTD is the fifth dose. For the proposed method, the dose levels are scaled by the transformation $(x+1.5)/2$, under which the MTD is 0.75. We set $s=3$ for the local modeling throughout.

**Fig. 1:**
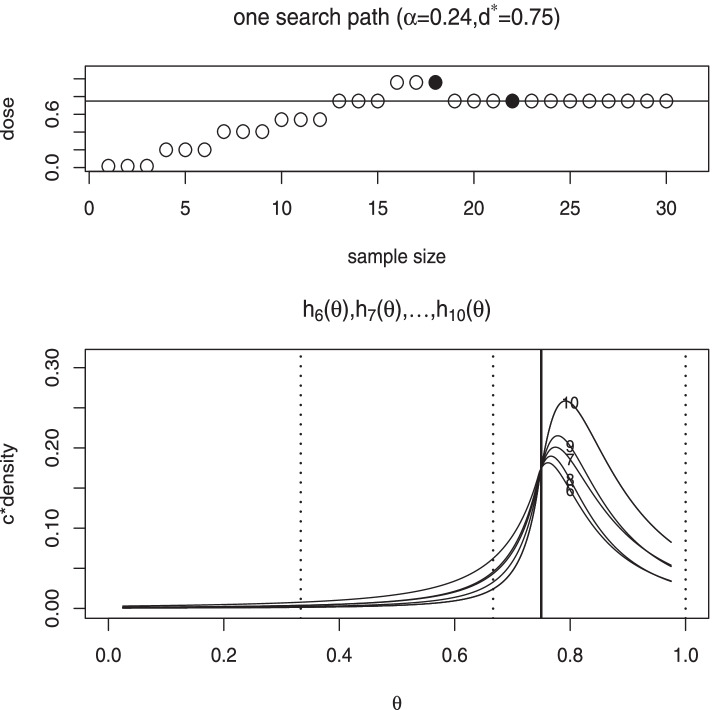
Upper panel: one search path up to 10 steps for the target toxicity probability 0.2, where the observed values of $y_n$ are depicted along the dose level by empty circles for 0 and filled bullets for 1. Lower panel: evolution of the posterior densities (multiplied by a certain constant for illustration purpose) of $\theta$ after the sixth to tenth cohort. The dotted lines indicate the limits of the subintervals and the solid line indicates the root at 0.75

The upper panel of Fig. 1 shows one search path, (1, 2, 3, 4, 5, 6, 5, 5, 5, 5), obtained by the proposed method. It is seen that the fast escalation proceeded until the first DLT was observed on the 18th patient in the sixth cohort. Then, the design started to build a local model on the subinterval (0.667, 1) containing six data points. The posterior mean of $\theta$ after the sixth cohort was 0.729, which led to the fifth dose level in the same subinterval. In the seventh cohort, zero DLT was observed and the posterior mean of $\theta$ was 0.776, which led to the fifth dose level again. In the eighth cohort, one DLT was observed and the posterior mean dropped to 0.760, which still indicated to stay. The subsequent ninth and tenth cohorts did not observe any DLT. And the posterior means increased to 0.791 and 0.814 consecutively, which led to remain at the fifth dose level as desired. The lower panel of Fig. 1 presents the evolution of corresponding posterior densities of $\theta$ after the sixth to tenth cohorts. It is seen that the posterior density after observing zero DLT in the seven cohort shifted more mass toward right and it shifted more mass toward left after encountering one DLT in the eight cohort. The posterior densities after the ninth and tenth cohorts gradually shifted toward right again as no DLT was observed. Overall, we see the search path was stable with one oscillation and had a quick convergence. The search pathes by the other methods are also provided in the supplementary materials.

### Incorporating historical information

When historical information is available, it is desired to have design to incorporate it in a sensible way. Zhou et al. [24] proposed a unified framework to incorporate informative prior information using the skeleton and prior effective sample size (PESS) and recommended the method iBOIN. Duan et al. [25] proposed the method of Hi3+3 that first determines PESS from historical information through the power prior approach and then applies the i3+3 procedure [26] to the combined data.

Here, we extend the proposed method to incorporate historical information after Zhou et al. [24]. For $k=1,\ldots ,K$, let $p_k=\pi (d_k)$. Denote the elicited prior probability of $p_k$, i.e. skeleton (as in CRM), by $q_k$. Let $n_{0k}$ denote the PESS at dose level $d_k$. To determine PESS, we follow the two rules of thumb [24]. First, if there is strong evidence that the prior is correctly specified, choose large PESS to borrow more information to increase the accuracy and reliability. Second, if the prior is vague, set $n_{0k}$ to be an integer in [*N*/(3*K*), *N*/(2*K*)], where *N* is maximum sample size allowed. For example, when $N=30$, we set $n_{0k}=3$ or 2 for $K=5$ or 6, respectively. Let $a_k$ and $b_k$ be the nearest integers of $n_{0k}q_k$ and $n_{0k}(1-q_k)$, respectively. For instance, when $q_k=0.3$ and $n_{0k}=3$, $a_k=1$ and $b_k=2$. Then, we simply assign $a_k$ DLT events and $b_k$ non-DLT events as pre-existing observations at dose $d_k$ in Step 2 of the algorithm. These $a_k+b_k$ observations and the subsequent observations at $d_k$ are combined to compute the likelihood function. We refer this modified algorithm as hBSA.

### Comparisons and evaluation metrics

We compare the proposed BSA method with six popular methods, namely, 3+3 [3], CRM [6], mTPI [27], mTPI-2 [11], BOIN [13] and Keyboard [12]. The 3+3 method is carried out by the R package UBCRM [28]. The CRM, mTPI, mTPI-2, and BOIN are carried out by the commercial software ‘East BAYES’ with default settings, which are noted with superscript 1. In addition, the CRM, BOIN and Keyboard are carried out by the free softwares at trialdesign.org, which are noted with superscript 2.

We use the following four metrics to evaluate the performance of the designs.

I: the percentage of correct selection (PCS)
II: the percentage of patients allocated to the MTD (MTD%)
III: the percentage of patients treated above the MTD (above-MTD%)
IV: the average number of observed DLTs throughout the trial (# of DLTs)

Both PCS and MTD% reflect the accuracy of the estimation. The latter also reveals the speed of convergence to the MTD. Metrics III and IV evaluate the safety in terms of overdose control and overall cost of adverse event, respectively. Throughout, we set the maximum sample size to be 30 in ten cohorts of size three and set the number of replications to be 10,000.

## Results

### Fix scenario case

We adopt the 20 representative scenarios of toxicity rates from Yan et al. [12] in Table S1, where the target toxicity rates are 20% for the first 10 scenarios and 30% for the last 10 scenarios, such that the MTD is located from low level to high level out of five doses. We first convert the dose levels to 0.1, 0.3, 0.5, 0.7, 0.9 as described earlier and set the number of subintervals *s* to be three throughout. Sensitivity study about *s* is given later.

**Fig. 2:**
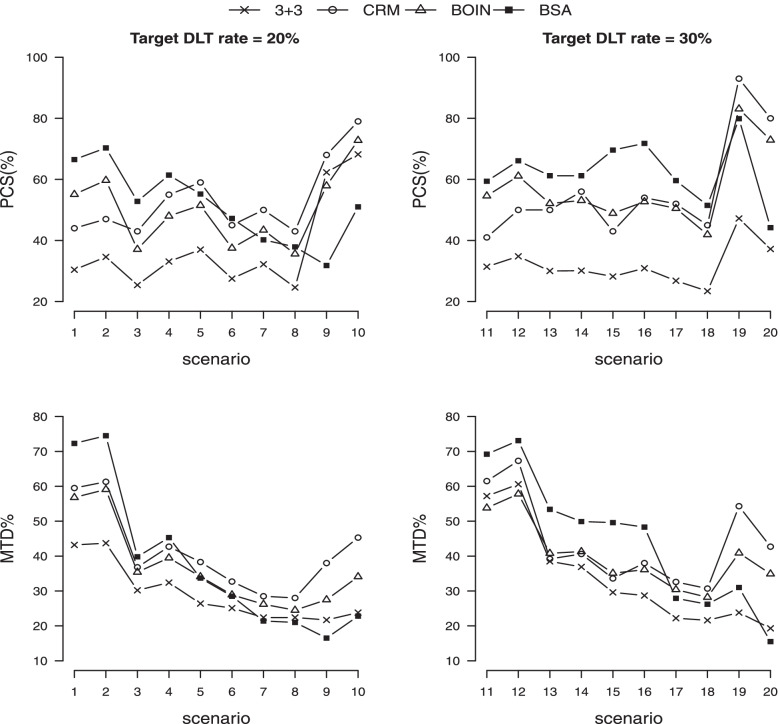
PCS and MTD% obtained by four competing designs. A higher value is better

**PCS of MTD** Figure 2 shows the comparison of PCS obtained by 3+3, CRM$^2$, BOIN$^2$ and BSA. (The values of the number of DLTs are replaced by the counterparts from CRM$^1$, BOIN$^1$ since they are not available from the freeware.) The results from the commercial software (mTPI, mTPI-2, CRM$^1$, BOIN$^1$) are either close to their respective counterparts by freeware or uniformly inferior to BOIN$^2$. The results by Keyboard are found very close to those by BOIN$^2$, as reported by Yan et al. [12]. Therefore they are excluded in the graphical comparison. The complete results of all seven competing methods are given in Table S2.

The 3+3 method exhibits its inefficiency with average PCSs of 37.5% and 32.0% for $\alpha =20\%$ and 30%, respectively. Under $\alpha =20\%$, the proposed BSA method yields the highest PCS in five scenarios (sc 1–4 and 6), where the MTD is located in lower levels. The superiority margin to the second best is as wide as 10%. The CRM$^2$ produces the highest PCS in the remaining five scenarios (sc 5, 7–10) where the MTD is located in higher levels. In average, the PCS of the best two performers BSA and CRM$^2$ are 51.4% and 53.3%, respectively. These reveal the conservativeness of BSA pointed out before especially when the target DLT probability is small and the MTD is late positioned. Under $\alpha =30\%$ which is most common in practice, BSA yields the highest PCS (all above 50%) in the first eight scenarios (sc 11–18), where the MTD is located in the first four levels. The superiority margin to the second best varies from 3% to 20%. This shows a remarkable improvement over the existing methods under the most representative scenarios. The CRM$^2$ produces the highest PCS in the remaining two scenarios (sc 19–20) where the MTD is located in the highest (fifth) level. In average, the PCS of the best two performers BSA and CRM$^2$ are 62.5% and 56.4%, respectively. At last, we note that scenarios 9, 10, 19, 20 with MTD at the last level are rare in reality as clinician would propose a search domain elicited from historical trials or PK/PD studies and specify candidates so that the guessed MTD is not close to the end of the search window.

**MTD%** The results of MTD% are in alignment with those of PCS. The best performer with the highest value is either BSA for the lower level MTD case (sc 1–4 under $\alpha =20\%$ and sc 11–16 under $\alpha =30\%$) or CRM$^2$ for the higher level MTD case (sc 5–10 under $\alpha =20\%$ and sc 17–20 under $\alpha =30\%$). See complete results in Table S3.

**Fig. 3:**
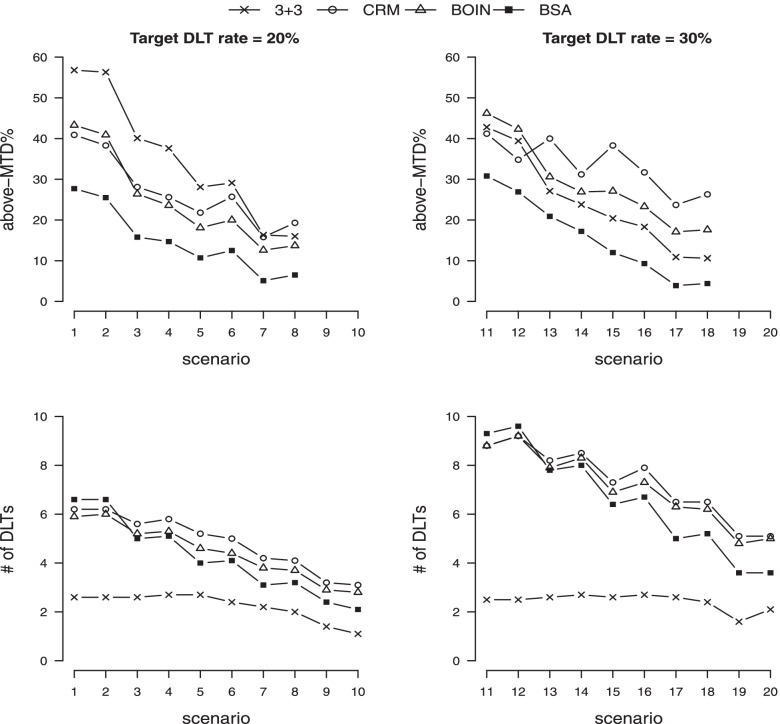
Above-MTD% and # of DLTs obtained by four competing designs. A lower value is better

**above-MTD%** Besides the excellent performance of BSA in estimation accuracy, its advantage in overdose control is more pronounced. Figure 3 and Table S4 show that BSA yields the lowest above-MTD% among seven competing methods uniformly across 20 scenarios. The averaged improvement margin to the second best is about 6%. This is resulted from the conservativeness and monotonic search path of BSA as illustrated before. On the other hand, the methods like CRM and BOIN are more library in dose escalation.

**number of DLTs** We note that the comparison with 3+3 should not be accounted as its known tendency to underestimate. The proposed BSA method yields the minimum number of DLTs under 16 scenarios (sc 3–10 and 13–20) when the MTD is not at the first level (out of six competing methods excluding 3+3). When the MTD is at the first level (in scenarios 1–2 and 11–12), the performance of BSA is comparable to the others within maximum 0.7 number of DLTs in average. See complete results in Table S5.

**quick actions by BSA** The quick actions of BSA described in Section 2.2 depends on the actual probability of toxicity. For instance, in scenarios 9, 10, 19 and 20, where the probabilities of toxicity are rather small in the early doses and the MTD is located at the last dose, the quick actions invoked by either zero DLT event (2.49$\sim$3.73 times out of ten) or Wald-type interval (0.64$\sim$0.99 times out of ten) take more places than in the other scenarios. We emphasize that the hybrid of these frequentist actions with the proposed Bayesian method saves computational cost and has nearly no impact to the estimation accuracy. The detailed results are given in Table S6.

**performance with the use of historical information** For the sake of space, we defer the comparison of the proposed hBSA with iBOIN in the presence of historical information to Supplementary Materials. In summary, the proposed hBSA outperforms iBOIN in estimation accuracy in average when the skeleton is correctly specified. The superiority is more prominent when the MTD is at the early or middle position of the search domain. When the skeleton is mis-specified, hBSA performs comparable to iBOIN with superiority for MTD at early or middle position and inferiority for MTD at late position. hBSA has significant better overdose control than iBOIN no matter the skeleton is correctly specified or mis-specified.

### Random scenario case

Now fix the target rate at 30%. We repeat the comparison in Section 3.1 under more scenarios that are randomly generated. We adopt the pseudo-uniform algorithm by Clertant et al. [29] to generate 200 random scenarios (of the probabilities of toxicity) for $K=5$ and 6 respectively with the MTD equally probably located at the first four doses, where the toxicity probability gap between the MTD and its adjacent doses is within (0.05, 0.3) [24]. Twenty random scenarios for $K=5$ and 6 are shown in Fig. S4.

For the proposed BSA method, we first use the same dose levels (derived from the ranks) as in Section 3.1, referred as ‘BSA (fixed)’. Second, to examine the performance of using exact dose information, we randomly generate dose levels. To be specific, we first set the MTD, say $d_k$, at 0.3, 0.5, 0.7 to represent the early, middle and late position in the search domain, respectively, and then generate $k-1$ doses in $(0,d_k-0.05)$ and $K-k$ doses in $(d_k+0.05,1)$, where the vicinity $(d_k-0.05,d_k+0.05)$ of MTD is created to make it distinguishable from other doses. We refer BSA under these three settings as ‘BSA (exact, early)’, ‘BSA (exact, middle)’ and ‘BSA (exact, late)’, respectively. Note that the other competing methods do not distinguish such location specifications since only ranks are used.

**Fig. 4:**
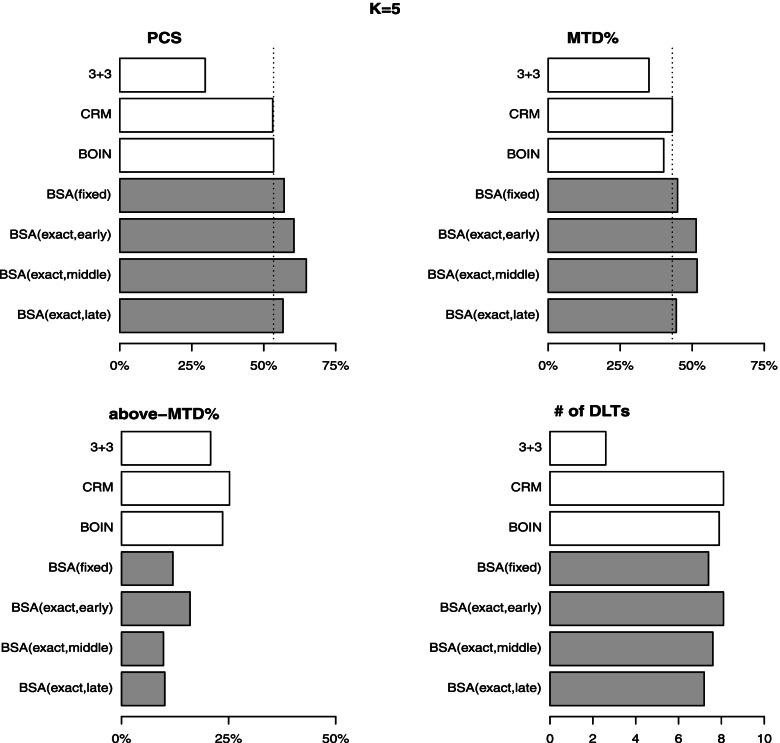
Performance of four competing designs under random scenarios with $K=5$

**Fig. 5:**
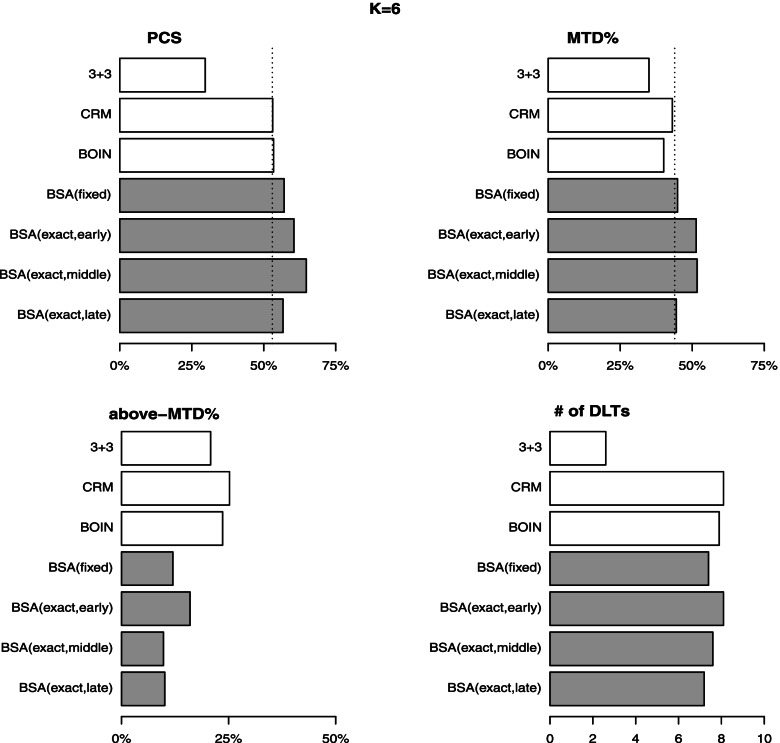
Performance of four competing designs under random scenarios with $K=6$

Figures 4 and 5 compare the performance (in four metrics averaged over 200 random scenarios) of 3+3, CRM$^2$, BOIN$^2$ and BSA. The complete report of all seven methods is given in Table S13. In general, the results are seen consistent to the findings of the fixed scenario case. Regarding the estimation accuracy, the 3+3 method performs the worst with the average PCS below 30% for both $K=5$ and 6. The mTPI and mTPI-2 produce the average PCS around 47–51%. The CRM, BOIN, and Keyboard perform comparably with the average PCS around 53% ($\pm 1\%$) (for both $K=5$ and 6). The proposed BSA (fixed) produces improved results with the average PCS of 57.1% and 58.0% for $K=5$ and 6, respectively. The BSA (exact, early) yields the average PCS of 60.5% and 58.0% for $K=5$ and 6, respectively. The BSA (exact, middle) yields the average PCS of 64.8% and 64.4% for $K=5$ and 6, respectively, implying a minimum 10% superiority lead to the other competing methods. The BSA (exact, late) yields the average PCS of 56.7% and 56.8% for $K=5$ and 6, respectively, which still outperforms the other competing methods. The outcomes of the second accuracy metric, MTD%, show consistent results with those of PCS. The proposed BSA method (either fixed or exact) outperforms the other competing methods with the largest values of MTD% under both $K=5$ and 6. It is noted that the results about accuracy of the other six competing methods are consistent with the findings reported in Zhou et al. [2]. Regarding the other two metrics about safety, the proposed method demonstrates significant superiority to the other six methods with a reduction in overdose (above-MTD) rate varying in 3-10% from the second best performer depending on the location of MTD. The proposed method controls the number of DLTs comparably as the other methods after excluding the conservative 3+3 method.

Again, we defer the results of the comparison with iBOIN in the presence of historical information to Supplementary Materials. The findings are similar to those from the fixed scenarios.

### Sensitivity analysis

We first conduct sensitivity analyses for the proposed method with respect to the number of subinterval *s* by repeating the simulation in Section 3.1. Recall that *s* determines the length of subintervals based on which the most recent data are collected and the local model is built. Tables S15 and S16 report the four metrics of BSA with *s* set to be 5 and 7, respectively. It is seen that the performance are comparable to those under $s=3$ (Tables S2-S5) with a slight variation within 1% in average PCS. In general, the number of subinterval regulates the quantity of local information to be used for transition decision making. Smaller value of *s* implies larger subinterval or more local data points, which leads to more conservative transition action. Therefore, it is in favor of the scenario with MTD in early position. On the other hand, larger value of *s* results in more aggressive decision making and favors the scenario with MTD in late position. To balance the relevancy and accuracy, we recommend setting $s=3$ (with the subinterval width of 1/3) for $K\le 6$ and $s=5$ (with the subinterval width of 1/5) for $K\ge 7$ in practice. Of course, for large *K* more sample size is needed.

Second, we examine the impact of cohort size. We set the cohort size to be one (i.e., full sequential design), two and a randomly varying number in $\{1,2,3\}$, respectively, while keeping the maximum sample size to be 30. (So that the corresponding numbers of cohort become 30, 15 and a number between 10 and 30, respectively.) Such settings make the design more liberal as fewer samples are needed to make a move at each cohort. We carry out the BSA design with these settings under the same 20 representative scenarios. Tables S17 and S18 show that in contrast to the design with fixed cohort size of three, the performance with smaller cohort sizes and varying cohort size in $\{1,2,3\}$ improves in accuracy, especially for the scenarios where the MTD resides at a late position and declines sensibly as necessary scarification in overdose control, which is still superior to the other competing methods in average. This suggests that the proposed design is well suitable for flexible accrual.

Third, we repeat the simulation in Section 3.2 with the MTD randomly assigned to all *K* doses. In parallel to Tables S13 and S14, Tables S19 and S20 report the four metrics in comparison with the respective competing methods in the absence and presence of historical information. It is seen that the results are similar to those when MTD is in the first four doses.

## Discussion

We have proposed a novel dose-finding design for phase I clinical trials. The design employs the Bayesian stochastic approximation method that (i) takes the dose level information into account to improve the estimation efficiency and (ii) requires no pre-specifications of parameters. One remarkable advantage of the proposed design is that it allows varying cohort size throughout the trial. This flexibility enables the design to accommodate different accrual situations, e.g., to use small cohort size for slow accrual and a large cohort for fast accrual.

By comparing with some popular methods through either commercial software or free softwares, we show that based on a sample of 30 subjects the proposed BSA design produces the estimation accuracy with PCS of $\sim$60% for the common case of MTD at an early or middle level. And it is much safer in overdose control. The computation of the new design is simple. We provide a free online package to facilitate the application, which pre-prints the decision rules graphically. See an illustration of the use of the R Shiny application in Supplementary Materials. The R code for simulation is provided in Supplementary Materials as well.

Like the other designs for estimating MTD, the proposed design assumes the monotonicity of the dose-toxicity relationship. Therefore, it is not applicable for optimal biological dose where the assumption does not necessarily hold.

At last, we note that extensions of the design to handle combination drugs and to incorporate covariates such as regional or ethnic effects are worth further investigation.

## Supplementary Information

**Additional file 1:** Supplementary Materials.

## Data Availability

All data generated or analysed during this study are included in this article and its supplementary information file.
