# Supplementary material for: A dose-finding design for phase I clinical trials based on Bayesian stochastic approximation
Source: BMC Med Res Methodol. 2022 Oct 1;22:258. doi: 10.1186/s12874-022-01741-3 (PMC9526928; doi:10.1186/s12874-022-01741-3)
Supplement: Supplementary file 1 — Additional file 1: Supplementary Materials. [file 12874_2022_1741_MOESM1_ESM.pdf]

Supplementary Materials
"A dose-finding design for phase I clinical trials based on Bayesian stochastic approximation"

Jin Xu[1,2,], Dapeng Zhang[1], and Rongji Mu[3]

[1]School of Statistics, East China Normal University, Shanghai, China
[2]Key Laboratory of Advanced Theory and Application in Statistics and Data Science - MOE, East China Normal University, Shanghai, China
[3]Clinical Research Center, Shanghai Jiao Tong University School of Medicine, Shanghai, China

Corresponding author: Jin Xu, School of Statistics, East China Normal University, 3663 North Zhongshan Road, Shanghai 200062, China, email: jxu@stat.ecnu.edu.cn

A Methods

A.1 Design

A.1.1 Transformation of dose levels

A general rule to transform the dose levels in $(0, 1)$ is as follows.

1) Set the search domain by $[a, b] = [d_1 - 0.5(d_2 - d_1), d_K + 0.5(d_K - d_{K-1})]$ so that the dose levels are well spread in the range.

2) Convert the dose levels in $(0, 1)$ by transformation $(d_k - a)/(b - a)$ for $k = 1, \ldots, K$.

If the original dose levels increase in fold change such as 100, 200, 400 and so on. Do logarithm transformation before Step 1.

A.1.2 Illustration of the local modeling

Figure S1 illustrates the approximation of $\pi(x)$ in (v_0, v_1) containing x_n by a line segment.

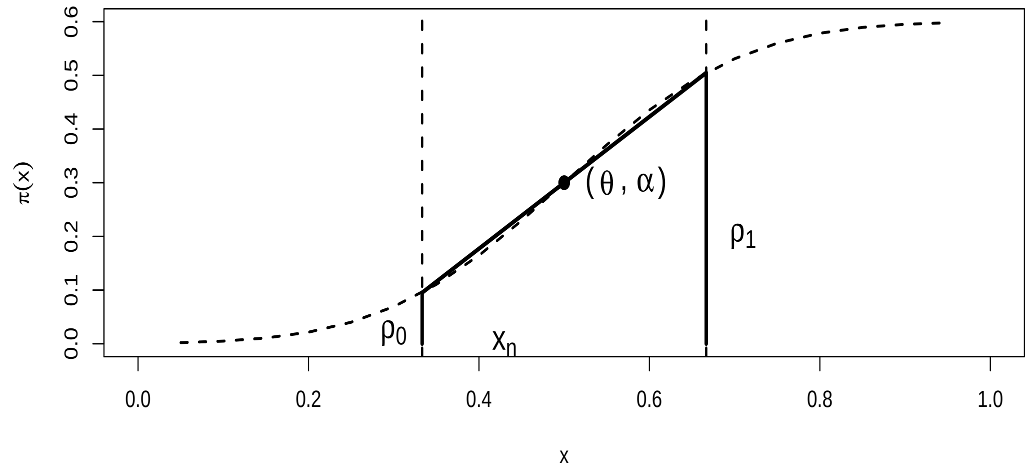

Figure S1: Illustration of the approximation of $\pi(x)$ in (v_0, v_1) containing x_n by a line segment

A.1.3 Property of coherence

Proposition 1. *The proposed design is coherent in the sense that the probability of dose escalation (or de-escalation) is 0 when the DLT response is 1 (or 0) at the current dose.*

Proof. Our proof follows closely with Cheung [1].

Suppose that the subinterval determined by the current dose contains m points, denoted by $\mathcal{D}_m = \{(x_1, y_1), \ldots, (x_m, y_m)\}$. Without loss of generality, suppose that (x_m, y_m) is the last observation. Denote $\mathcal{D}_{m-1} = \{(x_1, y_1), \ldots, (x_{m-1}, y_{m-1})\}$.

To establish the coherence under escalation, we need to show $\mathrm{E}(\theta|\mathcal{D}_m) \leq \mathrm{E}(\theta|\mathcal{D}_{m-1})$ if $y_m = 1$, which leads to no escalation by (1). Let ℓ_m denote the likelihood of the m points in the subinterval. Let $\pi(\theta|\beta)$ denote the induced prior distribution function of θ given β. Let $\pi(\beta)$ denote the induced marginal prior distribution function of β. Observe that

$$\begin{aligned}
&\mathrm{E}(\theta|\mathcal{D}_m) - \mathrm{E}(\theta|\mathcal{D}_{m-1}) \\
=&\mathrm{E}_\beta \left\{ \mathrm{E}^{(m)}(\theta|\beta) - \mathrm{E}^{(m-1)}(\theta|\beta) \right\} \\
=&\int \left\{ \frac{\int \theta \ell_m(\theta,\beta) d\pi(\theta|\beta)}{\int \ell_m(\theta,\beta) d\pi(\theta|\beta)} - \frac{\int \theta \ell_{m-1}(\theta,\beta) d\pi(\theta|\beta)}{\int \ell_{m-1}(\theta,\beta) d\pi(\theta|\beta)} \right\} d\pi(\beta) \\
=&\int \frac{\int \theta \ell_m(\theta,\beta) d\pi(\theta|\beta) \int \ell_{m-1}(\theta,\beta) d\pi(\theta|\beta) - \int \theta \ell_{m-1}(\theta,\beta) d\pi(\theta|\beta) \int \ell_m(\theta,\beta) d\pi(\theta|\beta)}{\int \ell_m(\theta,\beta) d\pi(\theta|\beta) \int \ell_{m-1}(\theta,\beta) d\pi(\theta|\beta)} d\pi(\beta).
\end{aligned} \tag{S1}$$

Since $\ell_m(\theta,\beta) = F(x_m;\theta,\beta)\ell_{m-1}(\theta,\beta)$ when $y_m = 1$, the numerator of the integrant in (S1) can be expressed as

$$N = \int\int \gamma \ell_{m-1}(\gamma,\beta)\ell_{m-1}(\theta,\beta)\{F(x_m;\gamma,\beta) - F(x_m;\theta,\beta)\} d\pi(\gamma|\beta) d\pi(\theta|\beta). \tag{S2}$$

By symmetry,

$$N = \int\int \theta \ell_{m-1}(\theta,\beta)\ell_{m-1}(\gamma,\beta)\{F(x_m;\theta,\beta) - F(x_m;\gamma,\beta)\} d\pi(\theta|\beta) d\pi(\gamma|\beta). \tag{S3}$$

Adding (S2) and (S3) yields

$$2N = \int\int \ell_{m-1}(\gamma,\beta)\ell_{m-1}(\theta,\beta)(\gamma-\theta)\{F(x_m;\gamma,\beta) - F(x_m;\theta,\beta)\} d\pi(\gamma|\beta) d\pi(\theta|\beta) < 0,$$

since $(\gamma-\theta)\{F(x_m;\gamma,\beta) - F(x_m;\theta,\beta)\} < 0$. After confining to the candidate set $\mathcal{C}$ through (1), we get $\mathrm{E}(\theta|\mathcal{D}_m) \leq \mathrm{E}(\theta|\mathcal{D}_{m-})$.

Similarly, we can show the coherence under de-escalation.

□

A.2 Example

Figure S2 shows the search paths by five other methods given the same responses of DLT as in Figure 1 for the proposed method. The search path by CRM deviates from the 7th cohort as $(6, 6, 5, 6)$; the search path by mTPI deviates from the 7th cohort as $(6, 6, 6, 6)$; the search pathes obtained by mTPI-2, BOIN and Keyboard deviate from the 8th cohort as $(6, 6, 6)$. All these five methods lead to one level above the target (fifth) dose indicated by the horizontal line.

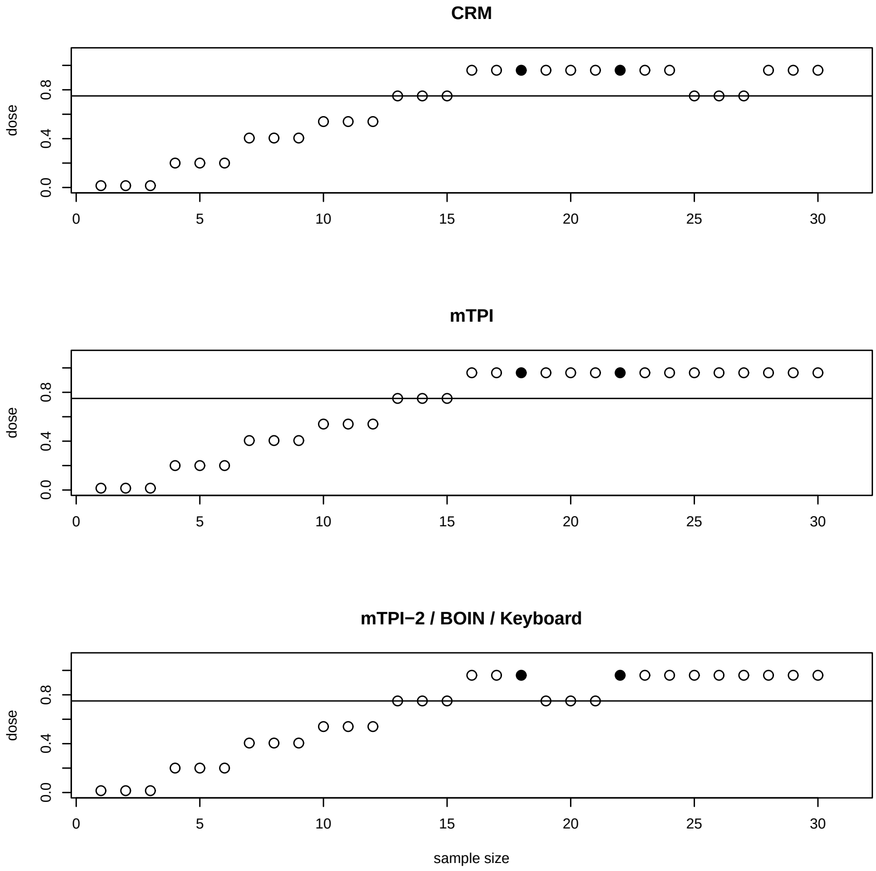

Figure S2: Given the dose-toxicity model in Example and the same responses of DLT as in Figure 1, the upper panel shows the search path obtained by CRM, the middle panel shows the search path obtained by mTPI, and the lower panel shows the same search path obtained by mTPI-2, BOIN and Keyboard. The target (fifth) dose is indicated by the horizontal line.

B Results

B.1 Fix scenarios case

B.1.1 without historical information

Table S1 contains the 20 representative scenarios of toxicity rates from Yan et al. [2], where the target toxicity rates are 20% for the first 10 scenarios and 30% for the last 10 scenarios. The MTD is located from low level to high level out of five doses.

Tables S2-S5 report the complete results of the four metrics obtained by all seven competing methods. The 3+3 method is carried out by the R package `UBCRM` [3]. The CRM, mTPI, mTPI-2, and BOIN are carried out by the commercial software 'East BAYES' with the default settings, which are noted with superscript 1. In addition, the CRM, BOIN and Keyboard are carried out by the free software at `trialdesign.org`, which are noted with superscript 2.

Table S6 reports the average numbers of the two types of quick actions (i.e., escalation in the absence of DLT and escalation/de-escalation by Wald interval) under 20 scenarios given in Table S1, where the action led by Wald interval is decomposed in escalation and de-escalation.

B.1.2 with historical information

We compare the proposed hBSA with iBOIN in the presence of historical information. The same specification of PESS is used by both methods. (The comparison with Hi3+3 is not included as it specifies PESS in a different way.) The iBOIN is carried out by free software at `trialdesign.org`.

In addition to the setup of the probabilities of toxicity in Table S1, two kinds of skeletons are specified in Tables S7-S8. The first kind matches with the true probabilities of DLT at MTD, called 'correctly specified skeleton'. The second kind mismatches with the true probabilities of DLT at MTD by one or two levels off (such as in Scenarios 2 and 1 respectively), called 'mis-specified skeleton', as described in A.3.2 of Zhou et al. [4].

Tables S9-S10 as well as Figures S5-S6 compare the two competing methods in three metrics under four combinations made from target toxicity rate and skeleton specification. (The number of DLTs is not reported by iBOIN at `trialdesign.org`.) When the skeletons are correctly specified, the proposed hBSA yields high accuracy with the average PCS of 69.7% under $\alpha = 20\%$ and 74.0% under $\alpha = 30\%$, which are respectively 11.7% and 6.3%

larger than those of iBOIN. Especially, it performs significantly better when the MTD is not in the last/fifth dose, as seen in the case without historical information. In terms of the second accuracy measure of MTD%, under $\alpha = 20\%$ hBSA produces the higher rates than iBOIN when the MTD is at the early (first three) position and lower rates when the MTD is at the late (last two) positions, as being consistent to the performance in PCS. Under $\alpha = 30\%$, hBSA outperforms iBOIN uniformly across all cases with the average MTD% of 54.2% vs 46.1%. Regarding the safety measure of above-MTD%, hBSA shows superiority to iBOIN with significant margins for both $\alpha = 20\%$ and 30%. When the skeleton is mis-specified, hBSA performs comparable to iBOIN in estimation accuracy (with PCS 47.1% vs 49.2% on average and with MTD% 38.1% vs 35.5% on average) and better in overdose control (with above-MTD% 22.6% vs 30.8%).

Moreover, we repeat the comparison with the PESS doubled, i.e. $n_{0k} = 6$, to represent a prior with more historical data. So that when $q_k = 0.3$, we have $a_k = 2$ and $b_k = 4$ proportionally. In parallel to Tables S9-S10, Tables S11-S12 report the comparison of the two competing methods in three metrics under four combinations made from target toxicity rate and skeleton specification with $n_{0k} = 6$. With more informative prior, the findings are consistent to those under the vague prior with PESS $n_{0k} = 3$. When the skeleton is correctly specified (Table S11), the superiority of hBSA to iBOIN is more pronounced. When the skeleton is mis-specified (Table S12), hBSA and iBOIN are comparable. In both situations, hBSA outperforms iBOIN in all three metrics in average.

B.2 Random scenario case

B.2.1 without historical information

Figure S4 shows twenty random scenarios of toxicity rates for $K = 5$ and 6 under the target rate 30%.

Table S13 reports the performance (in four metrics averaged over 200 random scenarios) of the seven competing methods for $K = 5$ and 6 respectively, where the MTD equally probably located at the first four doses.

B.2.2 with historical information

We compare the proposed method with iBOIN in the presence of historical information under 200 random scenarios under $K = 5$ and 6, respectively, where the prior of MTDs are specified in the same two ways as in Section 3.1. For the mis-matched skeleton, 50 scenarios

are randomly generated each for one or two levels off in two (opposite) directions. The complete results are given in Table S14.

When $K = 5$, the proposed method with fixed dose performs better than iBOIN in accuracy (w.r.t. both PCS and MTD%). The improvements are more pronounced by hBSA with exact dose information when the MTD is at early or middle position. The performance becomes inferior to iBOIN when the MTD resides at a late position, which is due to its conservatism noted before. These comparison results hold the same no matter the skeleton is correctly specified or mis-specified. When $K = 6$, hBSA outperforms iBOIN in PCS and MTD% under correctly specified skeleton. Under mis-specified case, hBSA performs inferior to iBOIN in MTD% when the MTD is at a late position. In all cases, hBSA yields significantly better overdose control than iBOIN regardless of the specification of the skeleton.

C Sensitivity analysis

C.1 Performance of BSA under different numbers of subintervals

Tables S15 and S16 report the performance of BSA using different number of subintervals s under the 20 representative scenarios in Table S1. It is seen that the performance are comparable to those under $s = 3$ (Tables S2-S5) with a slight variation between 1–2% in average PCS.

C.2 Performance of BSA under different cohort sizes

Tables S17 and S18 report the performance of BSA under the 20 representative scenarios in Table S1 with different number of cohort size. It is seen that the performance with smaller cohort sizes and varying cohort size in $\{1, 2, 3\}$ improves in accuracy, especially for the scenarios where the MTD resides at a late position, and declines sensibly as necessary scarification in overdose control, which is still superior to the other competing methods in average (Tables S2-S5).

C.3 Performance of BSA when the MTD is randomly assigned at all K doses

Table S19 reports the four metrics in comparison with the six competing methods as in Tables S13, where the MTD is randomly assigned at all K doses.

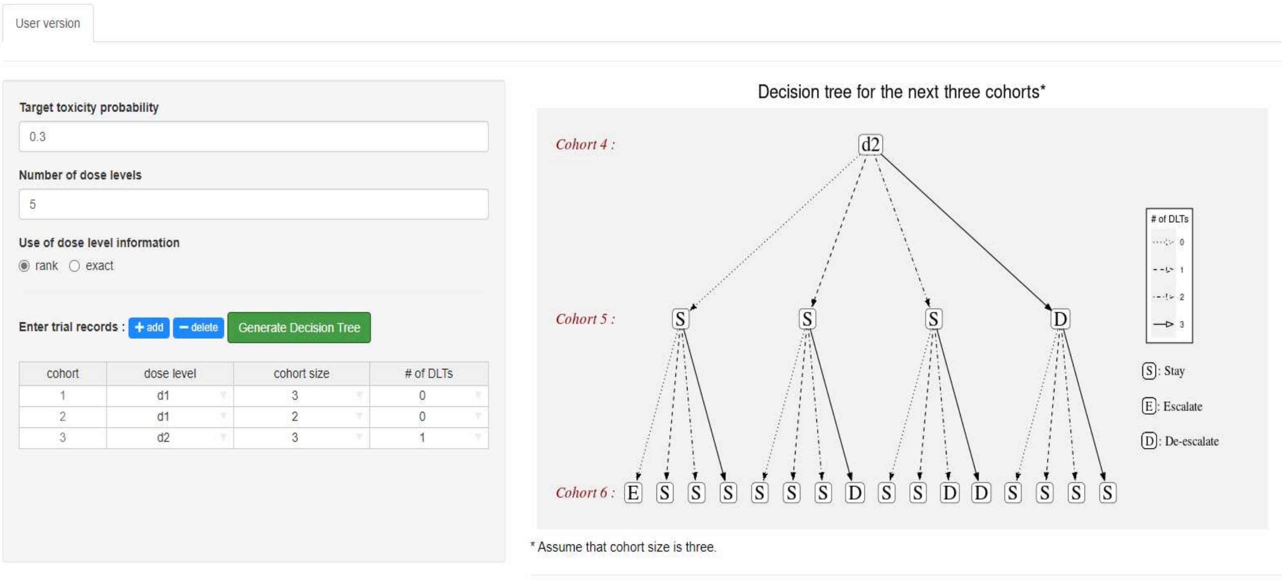

Figure S3: Illustration of an example on the online package

Table S20 reports the four metrics in comparison with the two competing methods as in Table S14 in the presence of historical information, where the MTD is randomly assigned at all K doses.

D Discussion

Figure S3 shows an example of the online package at `https://bsa4df.shinyapp.io/BSA_app`.

In this example, the user first inputs i) the target toxicity rate $\alpha = 0.3$, ii) the number of doses $K = 5$, iii) the option how to use the dose level information, where 'rank' stands for the fixed dose method. Then, the user inputs the records for up to the latest cohort, i.e., dose level, cohort size, observed number of DLTs for each cohort. Note that varying number of cohort size is allowed.

After clicking the 'Generate Decision Tree' button, the right panel displays a decision tree for transition action for the next three cohorts assuming the cohort size is three.

Table S1: Twenty representative toxicity scenarios in terms of true DLT rates for five doses, where the target DLT rates are 20% for the first ten scenarios and 30% for the last ten scenarios, respectively.

	target toxicity rate = 20%						target toxicity rate = 30%				
scen	d_1	d_2	d_3	d_4	d_5	scen	d_1	d_2	d_3	d_4	d_5
1	**0.20**	0.26	0.40	0.45	0.46	11	**0.30**	0.36	0.42	0.45	0.46
2	**0.20**	0.29	0.35	0.50	0.58	12	**0.30**	0.40	0.55	0.60	0.70
3	0.10	**0.20**	0.25	0.35	0.40	13	0.08	**0.30**	0.38	0.42	0.52
4	0.08	**0.20**	0.30	0.45	0.65	14	0.13	**0.30**	0.42	0.50	0.80
5	0.04	0.06	**0.20**	0.32	0.50	15	0.04	0.07	**0.30**	0.35	0.42
6	0.01	0.10	**0.20**	0.26	0.35	16	0.01	0.12	**0.30**	0.41	0.55
7	0.05	0.06	0.07	**0.20**	0.31	17	0.06	0.07	0.12	**0.30**	0.40
8	0.02	0.04	0.10	**0.20**	0.25	18	0.02	0.05	0.16	**0.30**	0.36
9	0.01	0.02	0.07	0.08	**0.20**	19	0.01	0.02	0.04	0.06	**0.30**
10	0.01	0.02	0.03	0.04	**0.20**	20	0.06	0.07	0.08	0.12	**0.30**

Table S2: PCS (%) obtained by the seven competing designs across 20 scenarios of Table S1, where the average is given in the last column

	sc1	sc2	sc3	sc4	sc5	sc6	sc7	sc8	sc9	sc10	ave
3+3	30.4	34.6	25.4	33.1	37.0	27.5	32.3	24.6	62.3	68.2	37.5
mTPI[1]	44.0	48.3	36.5	47.1	48.6	35.8	48.1	39.3	54.1	61.7	46.4
mTPI-2[1]	47.4	51.3	35.0	43.8	45.3	34.3	40.1	33.5	44.2	51.5	42.6
Keyboard[2]	54.8	57.8	36.5	47.1	52.1	38.5	42.8	35.1	57.9	72.8	49.5
CRM[1]	47.6	52.1	38.4	49.5	53.2	39.8	45.6	38.1	63.1	71.5	49.9
CRM[2]	44.0	47.0	43.0	55.0	59.0	45.0	50.0	43.0	68.0	79.0	53.3
BOIN[1]	47.5	52.4	41.4	51.1	53.0	40.4	46.5	37.7	56.7	69.8	49.7
BOIN[2]	55.1	59.7	37.1	48.0	51.5	37.5	43.4	35.6	57.9	72.8	49.9
BSA	66.5	70.3	52.8	61.4	55.2	47.3	40.2	37.9	31.8	51.0	51.4
	sc11	sc12	sc13	sc14	sc15	sc16	sc17	sc18	sc19	sc20	ave
3+3	31.4	34.8	30.0	30.1	28.2	30.9	26.8	23.4	47.2	37.2	32.0
mTPI[1]	45.5	53.9	47.8	52.3	43.2	50.8	49.6	43.2	68.7	61.4	51.7
mTPI-2[1]	43.1	51.7	42.4	48.0	37.5	46.1	46.2	39.9	64.5	61.5	48.1
Keyboard[2]	56.2	62.6	51.4	53.0	46.6	52.7	51.7	43.0	83.0	74.4	57.5
CRM[1]	47.8	57.3	43.7	52.4	37.7	48.9	47.8	40.6	83.7	72.7	53.3
CRM[2]	41.0	50.0	50.0	56.0	43.0	54.0	52.0	45.0	93.0	80.0	56.4
BOIN[1]	47.2	55.3	51.6	55.5	47.2	53.7	51.2	43.9	82.6	73.6	56.2
BOIN[2]	54.6	61.1	52.1	53.1	48.9	52.7	50.5	41.9	83.1	72.9	57.1
BSA	59.4	66.1	61.2	61.2	69.6	71.8	59.6	51.6	79.9	44.2	62.5

Table S3: MTD% obtained by the seven competing designs across 20 scenarios of Table S1, where the average is given in the last column

	sc1	sc2	sc3	sc4	sc5	sc6	sc7	sc8	sc9	sc10	ave
3+3	43.2	43.7	30.2	32.4	26.4	25.1	22.4	22.4	21.7	23.8	29.1
mTPI[1]	52.8	56.3	35.9	40.7	36.7	31.6	28.2	25.5	38.6	45.7	39.2
mTPI-2[1]	64.0	67.2	35.1	39.6	34.5	29.3	26.0	23.9	29.7	36.9	38.6
Keyboard[2]	56.3	57.7	35.3	39.2	35.0	29.3	26.0	24.5	27.2	34.3	36.5
CRM[1]	57.8	60.6	34.2	39.6	37.0	32.0	28.2	26.8	37.5	43.7	39.7
CRM[2]	59.5	61.3	36.8	42.7	38.3	32.7	28.5	28.0	38.0	45.3	41.1
BOIN[1]	63.4	66.6	35.5	39.8	34.6	29.4	26.1	23.9	29.7	36.9	38.6
BOIN[2]	56.8	59.1	35.4	39.5	34.1	28.9	26.2	24.5	27.5	34.1	36.6
BSA	72.3	74.5	39.8	45.3	33.7	28.5	21.4	21.0	16.5	22.8	37.6
	sc11	sc12	sc13	sc14	sc15	sc16	sc17	sc18	sc19	sc20	ave
3+3	57.2	60.6	38.5	36.9	29.6	28.7	22.2	21.6	23.8	19.3	33.8
mTPI[1]	60.4	64.3	47.1	46.8	40.9	41.3	32.5	30.5	48.7	37.8	45.0
mTPI-2[1]	59.6	64.2	42.9	43.7	36.9	38.4	31.1	29.2	46.1	37.8	43.0
Keyboard[2]	53.5	57.6	40.3	40.6	34.4	36.6	30.8	28.7	41.4	34.9	39.9
CRM[1]	61.0	66.3	39.1	41.2	35.3	38.2	31.9	30.6	50.5	38.9	43.3
CRM[2]	61.5	67.3	39.2	40.6	33.6	38.0	32.6	30.7	54.3	42.7	44.1
BOIN[1]	60.1	64.7	43.1	43.8	36.9	38.4	31.1	29.1	46.1	37.8	43.1
BOIN[2]	53.8	57.8	40.8	41.3	35.1	36.1	30.4	28.2	40.9	34.9	39.9
BSA	69.2	73.1	53.4	49.9	49.6	48.3	27.9	26.2	31.0	15.5	44.4

Table S4: above-MTD% obtained by the seven competing designs across 20 scenarios of Table S1, where the average (over first eight scenarios) is given in the last column (as above-MTD% is not applicable for the last two scenarios)

	sc1	sc2	sc3	sc4	sc5	sc6	sc7	sc8	sc9	sc10	ave
3+3	56.8	56.3	40.1	37.6	28.1	29.1	16.3	16.0	–	–	35.1
mTPI[1]	47.2	43.7	31.8	29.2	24.2	26.0	18.3	20.9	–	–	30.2
mTPI-2[1]	36.0	32.8	23.2	20.5	17.0	18.4	12.0	13.9	–	–	21.7
Keyboard[2]	43.6	42.4	26.9	24.2	18.7	20.2	13.1	15.1	–	–	25.5
CRM[1]	42.2	39.4	31.4	27.5	22.5	26.5	15.9	19.6	–	–	28.1
CRM[2]	40.9	38.3	28.1	25.6	21.8	25.7	15.8	19.3	–	–	26.9
BOIN[1]	36.6	33.4	23.3	20.6	17.0	18.4	12.0	13.9	–	–	21.9
BOIN[2]	43.3	40.9	26.4	23.6	18.1	20.0	12.6	13.7	–	–	24.8
BSA	27.7	25.5	15.8	14.7	10.7	12.5	5.1	6.5	–	–	14.8
	sc11	sc12	sc13	sc14	sc15	sc16	sc17	sc18	sc19	sc20	ave
3+3	42.8	39.4	27.1	23.8	20.4	18.3	10.9	10.6	–	–	24.2
mTPI[1]	36.6	33.4	23.3	20.6	17.0	18.4	12.0	13.9	–	–	21.9
mTPI-2[1]	36.0	32.8	23.2	20.5	17.0	18.4	12.0	13.9	–	–	21.7
Keyboard[2]	46.7	42.4	31.9	27.4	27.7	23.3	17.0	17.8	–	–	29.3
CRM[1]	42.2	39.4	31.4	27.5	22.5	26.5	15.9	19.6	–	–	28.1
CRM[2]	41.2	34.8	40.0	31.2	38.3	31.7	23.7	26.3	–	–	33.4
BOIN[1]	36.6	33.4	23.3	20.6	17.0	18.4	12.0	13.9	–	–	21.9
BOIN[2]	46.2	42.3	30.6	26.9	27.1	23.3	17.1	17.6	–	–	28.9
BSA	30.8	26.9	20.9	17.2	12.0	9.3	3.9	4.4	–	–	15.7

Table S5: number of DLT obtained by the seven competing designs across 20 scenarios of Table S1, where the average is given in the last column

	sc1	sc2	sc3	sc4	sc5	sc6	sc7	sc8	sc9	sc10	ave
3+3	2.6	2.6	2.6	2.7	2.7	2.4	2.2	2.0	1.4	1.1	2.2
mTPI[1]	6.3	6.4	5.7	6	5.3	5	4.3	4.1	3.3	3.2	5.0
mTPI-2[1]	5.9	6	5.1	5.3	4.6	4.4	3.8	3.7	2.9	2.8	4.4
Keyboard[2]	–	–	–	–	–	–	–	–	–	–	–
CRM[1]	6.2	6.2	5.6	5.8	5.2	5.0	4.2	4.1	3.2	3.1	4.9
CRM[2]	–	–	–	–	–	–	–	–	–	–	–
BOIN[1]	5.9	6.0	5.2	5.3	4.6	4.4	3.8	3.7	2.9	2.8	4.5
BOIN[2]	–	–	–	–	–	–	–	–	–	–	–
BSA	6.6	6.6	5.0	5.1	4.0	4.1	3.1	3.2	2.4	2.1	4.2
	sc11	sc12	sc13	sc14	sc15	sc16	sc17	sc18	sc19	sc20	ave
3+3	2.5	2.5	2.6	2.7	2.6	2.7	2.6	2.4	1.6	2.1	2.4
mTPI[1]	8.8	9.2	8.1	8.4	7.1	7.4	6.3	6.2	5.0	5.0	7.2
mTPI-2[1]	8.9	9.2	7.9	8.4	6.9	7.3	6.3	6.2	4.8	5.0	7.1
Keyboard[2]	–	–	–	–	–	–	–	–	–	–	–
CRM[1]	8.8	9.2	8.2	8.5	7.3	7.9	6.5	6.5	5.1	5.1	7.3
CRM[2]	–	–	–	–	–	–	–	–	–	–	–
BOIN[1]	8.8	9.2	7.9	8.3	6.9	7.3	6.3	6.2	4.8	5.0	7.1
BOIN[2]	–	–	–	–	–	–	–	–	–	–	–
BSA	9.3	9.6	7.8	8.0	6.4	6.7	5.0	5.2	3.6	3.6	6.5

Table S6: The average numbers of the two types of quick actions (i.e., escalation in the absence of DLT and escalation/de-escalation by Wald-type interval) under 20 scenarios given in Table S1, where the action made by Wald-type interval is decomposed in escalation and de-escalation.

	sc 1	sc 2	sc 3	sc 4	sc 5	sc 6	sc 7	sc8	sc 9	sc10
es. w/o DLT	0.74	0.76	1.30	1.34	2.15	2.19	2.51	2.99	3.75	4.30
es. by Wald	0.02	0.02	0.27	0.39	0.66	0.57	0.62	0.66	0.90	0.99
de-es. by Wald	0.18	0.23	0.16	0.21	0.17	0.13	0.09	0.09	0	0
es. w/o DLT	0.48	0.46	1.14	0.94	1.99	1.88	2.15	2.50	3.73	2.49
es. by Wald	0.05	0.04	0.39	0.41	0.35	0.35	0.57	0.52	0.77	0.64
de-es. by Wald	0.14	0.17	0.18	0.17	0.17	0.16	0.06	0.07	0	0

Table S7: Pre-specified skeletons for the target DLT rate of 20%

scen	d_1	d_2	d_3	d_4	d_5
	correctly specified skeleton				
1	0.20	0.30	0.40	0.50	0.60
2	0.20	0.36	0.45	0.55	0.65
3	0.05	0.20	0.26	0.30	0.35
4	0.10	0.20	0.30	0.40	0.50
5	0.05	0.10	0.20	0.30	0.40
6	0.05	0.08	0.20	0.30	0.35
7	0.01	0.05	0.10	0.20	0.30
8	0.02	0.06	0.12	0.20	0.30
9	0.01	0.03	0.05	0.07	0.20
10	0.02	0.04	0.06	0.08	0.20
	mis-specified skeleton				
1	0.10	0.15	0.20	0.40	0.50
2	0.10	0.20	0.30	0.40	0.50
3	0.05	0.10	0.20	0.30	0.40
4	0.05	0.08	0.10	0.20	0.50
5	0.05	0.10	0.15	0.20	0.55
6	0.05	0.20	0.30	0.40	0.55
7	0.01	0.03	0.05	0.07	0.20
8	0.01	0.05	0.20	0.30	0.40
9	0.10	0.15	0.18	0.20	0.40
10	0.05	0.10	0.17	0.20	0.30

Table S8: Pre-specified skeletons for the target DLT rate of 30%

scen	d_1	d_2	d_3	d_4	d_5
	correctly specified skeleton				
11	0.30	0.36	0.45	0.50	0.55
12	0.30	0.40	0.45	0.50	0.60
13	0.10	0.30	0.40	0.50	0.60
14	0.05	0.30	0.40	0.55	0.70
15	0.05	0.10	0.30	0.40	0.50
16	0.02	0.05	0.30	0.45	0.55
17	0.05	0.08	0.12	0.30	0.45
18	0.01	0.05	0.10	0.30	0.50
19	0.01	0.03	0.05	0.07	0.30
20	0.05	0.06	0.07	0.08	0.40
	mis-specified skeleton				
11	0.10	0.20	0.30	0.40	0.50
12	0.05	0.30	0.45	0.50	0.55
13	0.05	0.10	0.20	0.30	0.50
14	0.05	0.10	0.30	0.40	0.55
15	0.01	0.05	0.15	0.20	0.30
16	0.01	0.05	0.15	0.30	0.50
17	0.05	0.30	0.35	0.40	0.55
18	0.05	0.10	0.30	0.40	0.55
19	0.01	0.05	0.30	0.40	0.50
20	0.01	0.05	0.10	0.30	0.40

Table S9: Comparison of hBSA and iBOIN under the fixed scenarios of Table S1 in terms of PCS (%), MTD%, and above-MTD% in the presence of historical information under *correctly specified* skeleton, where the average is given in the last column

$\alpha = 20\%$	sc1	sc2	sc3	sc4	sc5	sc6	sc7	sc8	sc9	sc10	ave
						PCS (%)					
iBOIN	58.3	64.4	46.0	55.8	59.4	47.6	55.8	44.9	71.9	79.5	58.4
hBSA	80.2	82.2	65.7	73.4	76.6	64.5	66.0	58.2	56.1	73.6	69.7
						MTD%					
iBOIN	58.9	64.1	38.6	42.5	37.8	33.6	32.1	29.0	34.7	38.3	41.0
hBSA	80.3	81.8	45.5	50.5	41.1	35.4	29.7	28.6	19.5	26.0	43.8
						above-MTD%					
iBOIN	41.2	35.9	27.4	23.4	19.0	21.7	14.2	15.2	–	–	24.8
hBSA	19.7	18.2	14.0	12.8	9.9	11.8	4.7	5.7	–	–	12.1

$\alpha = 30\%$	sc 11	sc 12	sc 13	sc 14	sc 15	sc 16	sc 17	sc 18	sc 19	sc 20	ave
						PCS (%)					
iBOIN	61.3	68.6	63.3	66.2	59.8	67.0	65.0	58.4	86.4	80.9	67.7
hBSA	69.2	75.9	62.8	66.0	75.4	78.5	73.2	66.7	91.1	81.5	74.0
						MTD%					
iBOIN	60.8	65.2	47.6	49.1	41.3	43.2	38.6	36.6	43.3	34.9	46.1
hBSA	70.3	73.8	61.0	61.3	56.4	57.7	42.3	38.2	45.5	35.0	54.2
						above-MTD%					
iBOIN	39.2	34.7	25.6	23.4	23.2	20.2	15.8	16.3	–	–	24.8
hBSA	29.7	26.2	21.7	18.4	12.0	9.7	3.6	3.2	–	–	15.6

Table S10: Comparison of hBSA and iBOIN under the fixed scenarios of Table S1 in terms of PCS (%), MTD%, and above-MTD% in the presence of historical information under *mis-specified* skeleton, where the average is given in the last column

$\alpha = 20\%$	sc1	sc2	sc3	sc4	sc5	sc6	sc7	sc8	sc9	sc10	ave
						PCS (%)					
iBOIN	42.4	52.4	38.8	43.8	56.2	44.3	48.9	42.6	50.5	71.7	49.2
hBSA	40.9	68.8	59.5	50.1	69.8	40.0	75.2	34.8	11.3	20.7	47.1
						MTD%					
iBOIN	47.7	52.3	35.8	36.2	34.8	30.9	30.9	26.4	25.5	34.3	35.5
hBSA	37.6	71.6	58.3	51.2	49.7	26.4	39.0	20.6	11.5	14.8	38.1
						above-MTD%					
iBOIN	52.3	47.7	36.7	36.5	24.6	16.3	19.6	13.0	–	–	30.8
hBSA	62.4	28.4	22.3	30.9	13.7	11.1	6.4	5.3	–	–	22.6

$\alpha = 30\%$	sc 11	sc 12	sc 13	sc 14	sc 15	sc 16	sc 17	sc 18	sc 19	sc 20	ave
						PCS (%)					
iBOIN	39.7	46.7	41.1	51.2	37.3	50.4	59.3	50.4	74.6	72.4	52.3
hBSA	47.4	58.2	46.0	52.4	64.6	72.3	61.8	55.2	66.8	58.2	58.3
						MTD%					
iBOIN	40.5	45.1	34.5	38.6	28.8	33.3	29.3	29.6	33.9	32.9	34.7
hBSA	46.3	52.3	45.6	50.0	56.9	59.8	29.3	28.5	21.1	17.1	40.7
						above-MTD%					
iBOIN	59.5	54.9	43.6	38.1	40.1	33.7	10.2	11.5	–	–	36.5
hBSA	53.7	47.7	42.2	37.1	18.8	15.2	2.5	2.6	–	–	27.5

Table S11: Comparison of hBSA and iBOIN under the fixed scenarios of Table S1 in terms of PCS (%), MTD%, and above-MTD% in the presence of historical information under *correctly specified* skeleton with $n_{0k} = 6$, where the average is given in the last column

$\alpha = 20\%$	sc1	sc2	sc3	sc4	sc5	sc6	sc7	sc8	sc9	sc10	ave
						PCS (%)					
iBOIN	62.5	72.1	50.0	60.7	64.6	55.2	61.5	52.1	76.2	80.2	63.5
hBSA	79.4	82.2	61.2	71.6	81.5	77.0	62.9	54.5	75.6	87.0	73.3
						MTD (%)					
iBOIN	64.3	70.4	41.2	45.3	40.8	38.9	35.4	32.6	39.1	40.0	44.8
hBSA	78.2	80.1	61.7	60.8	48.3	49.7	34.0	32.7	32.1	40.9	51.9
						above-MTD (%)					
iBOIN	35.7	29.6	28.2	19.8	16.8	19.4	14.2	15.0	–	–	17.9
hBSA	21.8	19.9	20.7	12.5	9.9	12.1	4.9	5.9	–	–	10.8

$\alpha = 30\%$	sc11	sc12	sc13	sc14	sc15	sc16	sc17	sc18	sc19	sc20	ave
						PCS (%)					
iBOIN	63.7	71.1	66.2	69.7	62.6	70.2	69.3	66.7	86.9	82.4	70.9
hBSA	68.6	76.4	65.1	63.7	76.5	85.8	72.5	66.2	96.5	92.4	76.4
						MTD (%)					
iBOIN	61.9	66.2	48.8	50.2	42.0	44.3	39.5	38.2	43.5	36.5	47.1
hBSA	68.5	72.8	62.8	62.6	58.5	64.7	42.2	37.1	53.9	48.3	57.1
						above-MTD (%)					
iBOIN	38.0	33.7	24.8	22.7	22.7	19.3	16.1	16.9	–	–	19.4
hBSA	31.5	27.2	19.5	23.0	11.8	5.5	2.5	2.5	–	–	12.3

Table S12: Comparison of hBSA and iBOIN under the fixed scenarios of Table S1 in terms of PCS (%), MTD%, and above-MTD% in the presence of historical information under *mis-specified* skeleton with $n_{0k} = 6$, where the average is given in the last column

$\alpha = 20\%$	sc1	sc2	sc3	sc4	sc5	sc6	sc7	sc8	sc9	sc10	ave
						PCS (%)					
iBOIN	38.5	51.6	39.9	38.3	53.6	50.0	45.9	51.9	39.4	63.8	47.3
hBSA	58.7	65.2	53.3	45.8	73.4	66.8	69.7	39.4	21.2	38.7	53.2
						MTD (%)					
iBOIN	43.7	53.0	38.7	31.1	36.9	35.2	32.1	30.7	24.0	32.2	35.8
hBSA	55.3	58.3	56.7	49.9	42.2	35.1	44.5	22.4	12.3	18.1	39.5
						above-MTD (%)					
iBOIN	56.3	47.1	38.6	48.6	26.9	6.7	27.4	5.4	–	–	25.7
hBSA	44.7	41.7	25.5	38.2	16.8	11.1	12.5	5.3	–	–	19.6

$\alpha = 30\%$	sc11	sc12	sc13	sc14	sc15	sc16	sc17	sc18	sc19	sc20	ave
						PCS (%)					
iBOIN	30.7	43.8	33.0	47.5	27.0	45.3	61.0	56.5	54.8	70.4	47.0
hBSA	28.2	42.9	31.4	48.8	58.3	75.7	63.3	53.4	45.8	51.0	49.9
						MTD (%)					
iBOIN	33.8	44.1	30.3	38.7	25.7	33.3	31.2	32.9	32.5	33.3	33.6
hBSA	25.3	35.6	33.2	47.7	54.1	62.9	28.6	27.3	15.2	14.2	34.4
						above-MTD (%)					
iBOIN	66.3	55.9	53.1	42.8	47.5	38.3	6.7	7.4	–	–	31.8
hBSA	74.7	64.4	55.9	41.0	22.8	14.0	2.2	2.2	–	–	27.7

Table S13: Performance of the six competing methods in PCS, MTD%, above-MTD% and number of DLTs averaged over 200 random scenarios, where the MTD is randomly assigned at the first four doses.

	PCS (%)	MTD%	above-MTD%	# of DLTs
			$K = 5$	
3+3	29.7	35.0	20.8	2.6
mTPI[1]	50.5	44.0	19.8	7.9
mTPI-2[1]	48.7	42.8	20.7	7.9
Keyboard[2]	53.7	39.9	24.0	–
CRM[1]	53.3	43.5	22.1	8.1
CRM[2]	53.1	43.1	25.2	–
BOIN[1]	54.2	42.9	20.5	7.9
BOIN[2]	53.4	40.1	23.6	–
BSA (fixed)	57.1	44.9	12.0	7.4
BSA (exact, early)	60.5	51.4	16.0	8.1
BSA (exact, middle)	64.8	51.7	9.8	7.6
BSA (exact, late)	56.7	44.5	10.1	7.2
			$K = 6$	
3+3	29.7	36.3	22.4	2.7
mTPI[1]	49.7	44.9	20.7	8.0
mTPI-2[1]	47.7	43.7	21.5	8.0
Keyboard[2]	53.3	40.5	25.2	–
CRM[1]	52.6	44.0	24.3	8.3
CRM[2]	52.7	43.9	26.9	–
BOIN[1]	53.4	43.8	21.4	7.9
BOIN[2]	53.0	40.6	24.8	–
BSA (fixed)	58.0	46.8	15.6	7.8
BSA (exact, early)	58.6	51.8	17.2	8.2
BSA (exact, middle)	64.4	53.0	10.7	7.7
BSA (exact, late)	56.8	46.4	11.2	7.4

Table S14: Performance of hBSA and iBOIN in PCS, MTD%, above-MTD% and number of DLTs averaged over 200 random scenarios with historical information, where the MTD is randomly assigned at the first four doses.

	method	PCS (%)	MTD%	above-MTD%	# of DLTs
			correctly specified skeleton		
$K = 5$	iBOIN	64.2	40.9	16.4	–
	hBSA (fixed)	64.2	46.3	6.4	7.1
	hBSA (exact, early)	66.7	57.3	12.3	8.1
	hBSA (exact, middle)	71.9	52.9	4.1	7.2
	hBSA (exact, late)	61.6	38.4	2.6	6.4
$K = 6$	iBOIN	62.3	45.4	21.9	–
	hBSA (fixed)	67.8	54.9	6.1	7.4
	hBSA (exact, early)	63.2	56.8	13.8	8.1
	hBSA (exact, middle)	71.6	57.7	3.9	7.3
	hBSA (exact, late)	64.8	45.9	2.0	6.5
			mis-specified skeleton		
$K = 5$	iBOIN	53.4	33.5	18.2	–
	hBSA (fixed)	54.5	37.2	11.4	7.1
	hBSA (exact, early)	57.4	45.1	15.5	7.8
	hBSA (exact, middle)	61.7	42.0	8.7	7.1
	hBSA (exact, late)	51.3	29.8	8.8	6.5
$K = 6$	iBOIN	52.2	38.1	24.6	–
	hBSA (fixed)	52.8	36.7	16.5	7.4
	hBSA (exact, early)	54.2	44.0	19.0	8.0
	hBSA (exact, middle)	59.8	42.8	10.7	7.2
	hBSA (exact, late)	53.0	33.7	9.6	6.6

Table S15: PCS and MTD% obtained by BSA under the same settings as Table S2 with different values of s, where the average across ten scenarios is given in the last column

PCS (%)

s	sc1	sc2	sc3	sc4	sc5	sc6	sc7	sc8	sc9	sc10	ave
3	66.5	70.3	52.8	61.4	55.2	47.3	40.2	37.9	31.8	51.0	51.4
5	51.4	56.4	63.8	73.8	62.8	47.4	40.5	37.5	33.1	51.2	51.8
7	45.1	49.8	64.8	71.4	71.4	57.2	27.3	24.0	42.9	62.9	51.7
	sc11	sc12	sc13	sc14	sc15	sc16	sc17	sc18	sc19	sc20	ave
3	59.4	66.1	61.2	61.2	69.6	71.8	59.6	51.6	79.9	44.2	62.5
5	48.0	53.7	74.5	76.9	72.3	70.0	60.0	54.2	73.8	38.8	62.2
7	50.2	54.6	73.9	75.1	79.8	76.0	48.3	42.3	76.5	39.9	61.7

MTD%

s	sc1	sc2	sc3	sc4	sc5	sc6	sc7	sc8	sc9	sc10	ave
3	72.3	74.5	39.8	45.3	33.7	28.5	21.4	21.0	16.5	22.8	37.6
5	60.4	63.3	50.4	57.2	32.1	27.1	21.2	20.3	16.6	22.8	37.1
7	54.2	56.2	53.8	58.9	37.8	31.7	15.5	14.6	21.8	28.9	37.3
	sc11	sc12	sc13	sc14	sc15	sc16	sc17	sc18	sc19	sc20	ave
3	69.2	73.1	53.4	49.9	49.6	48.3	27.9	26.2	31.0	15.5	44.4
5	62.9	65.5	64.1	60.2	48.1	44.8	27.3	26.6	23.6	12.9	43.6
7	65.0	66.6	64.2	58.9	51.8	48.4	19.1	17.7	32.9	17.0	44.2

Table S16: above-MTD% and number of DLTs obtained by BSA under the same settings as Table S2 with different values of s, where the average across ten scenarios is given in the last column

	above-MTD%										
s	sc1	sc2	sc3	sc4	sc5	sc6	sc7	sc8	sc9	sc10	ave
3	27.0	24.9	15.7	14.7	10.7	12.5	5.1	6.5	–	–	14.6
5	38.6	35.9	15.5	13.5	10.5	12.0	5.4	6.9	–	–	17.3
7	45.8	43.8	16.7	15.8	8.8	10.4	7.0	9.2	–	–	15.7
	sc11	sc12	sc13	sc14	sc15	sc16	sc17	sc18	sc19	sc20	ave
3	29.5	25.9	20.9	17.2	12.0	9.3	3.9	4.4	–	–	15.4
5	35.2	32.9	16.6	13.0	12.1	9.7	3.5	3.8	–	–	15.9
7	35.0	33.4	16.5	13.2	8.3	6.6	5.6	6.3	–	–	12.5
	# of DLTs										
s	sc1	sc2	sc3	sc4	sc5	sc6	sc7	sc8	sc9	sc10	ave
3	6.6	6.6	5.0	5.1	4.0	4.1	3.1	3.2	2.4	2.1	4.2
5	6.8	7.0	5.3	5.4	3.9	4.1	3.1	3.1	2.3	2.0	4.3
7	6.9	7.2	5.5	5.7	4.0	4.2	3.0	3.1	2.5	2.3	4.4
	sc11	sc12	sc13	sc14	sc15	sc16	sc17	sc18	sc19	sc20	ave
3	9.3	9.6	7.8	8.0	6.4	6.7	5.0	5.2	3.6	3.6	6.5
5	9.3	9.7	8.2	8.1	6.3	6.5	4.8	5.1	3.1	3.5	6.5
7	9.3	9.7	8.1	8.1	6.3	6.5	4.5	4.9	3.7	3.6	6.5

Table S17: PCS and MTD% obtained by BSA under the same settings as Table S2 with different cohort size c, where 'v' represents randomly varying cohort size in $\{1, 2, 3\}$ and the average across ten scenarios is given in the last column

					PCS (%)						
c	sc1	sc2	sc3	sc4	sc5	sc6	sc7	sc8	sc9	sc10	ave
1	65.6	69.9	50.8	60.9	57.7	42.9	41.3	39.2	49.2	69.0	54.7
2	65.0	69.5	49.1	58.8	55.0	45.8	45.2	42.2	41.6	60.5	53.3
3	66.5	70.3	52.8	61.4	55.2	47.3	40.2	37.9	31.8	51.0	51.4
v	66.4	70.1	52.3	60.3	55.0	45.8	44.1	41.5	39.3	59.6	53.4
	sc11	sc12	sc13	sc14	sc15	sc16	sc17	sc18	sc19	sc20	ave
1	55.0	64.7	58.2	60.1	60.0	66.6	62.2	52.6	85.9	61.3	62.7
2	57.8	65.5	61.7	61.7	66.5	69.1	59.6	50.8	80.0	48.5	62.1
3	59.4	66.1	61.2	61.2	69.6	71.8	59.6	51.6	79.9	44.2	62.5
v	57.1	64.6	59.8	61.3	65.0	68.0	59.7	51.4	82.1	51.0	62.0
					MTD%						
	sc1	sc2	sc3	sc4	sc5	sc6	sc7	sc8	sc9	sc10	ave
1	64.1	68.2	38.5	46.1	29.5	21.1	30.7	30.8	29.2	36.9	39.5
2	67.1	69.7	37.8	44.4	35.1	27.9	29.6	28.8	22.1	29.4	39.2
3	72.3	74.5	39.8	45.3	33.7	28.5	21.4	21.0	16.5	22.8	37.6
v	67.6	70.2	37.9	44.0	33.6	27.1	28.6	27.8	22.0	29.4	38.8
	sc11	sc12	sc13	sc14	sc15	sc16	sc17	sc18	sc19	sc20	ave
1	59.2	67.7	44.9	45.0	44.1	46.5	39.9	36.5	48.5	33.4	46.6
2	66.8	71.6	51.3	48.3	51.1	49.9	29.7	27.1	38.0	21.6	45.5
3	69.2	73.1	53.4	49.9	49.6	48.3	27.9	26.2	31.0	15.5	44.4
v	65.5	69.8	49.3	48.2	46.9	47.4	33.4	31.2	38.8	23.2	45.4

Table S18: above-MTD% and number of DLTs obtained by BSA under the same settings as Table S2 with different cohort size c, where 'v' represents randomly varying cohort size in $\{1, 2, 3\}$ and the average across ten scenarios is given in the last column

above-MTD%

c	sc1	sc2	sc3	sc4	sc5	sc6	sc7	sc8	sc9	sc10	ave
1	35.9	31.8	22.7	17.1	17.7	25.1	10.0	12.8	–	–	21.6
2	32.9	30.3	22.0	18.2	15.4	19.4	7.3	9.4	–	–	19.3
3	27.7	25.5	15.8	14.7	10.7	12.5	5.1	6.5	–	–	14.8
v	32.4	29.8	22.1	18.7	15.0	19.5	7.8	9.6	–	–	19.4

	sc11	sc12	sc13	sc14	sc15	sc16	sc17	sc18	sc19	sc20	ave
1	40.8	32.3	31.8	24.8	26.0	18.0	8.9	10.6	–	–	24.1
2	33.2	28.4	24.1	19.9	14.3	10.9	5.6	6.4	–	–	17.8
3	30.8	26.9	20.9	17.2	12.0	9.3	3.9	4.4	–	–	15.7
v	34.5	30.2	26.1	20.4	18.4	14.1	6.2	6.9	–	–	19.6

# of DLTs

	sc1	sc2	sc3	sc4	sc5	sc6	sc7	sc8	sc9	sc10	ave
1	7.0	7.1	5.6	5.6	4.5	4.7	3.9	3.9	3.1	2.9	4.8
2	6.8	6.9	5.4	5.4	4.5	4.5	3.7	3.7	2.7	2.4	4.6
3	6.6	6.6	5.0	5.1	4.0	4.1	3.1	3.2	2.4	2.1	4.2
v	6.9	6.9	5.4	5.5	4.5	4.5	3.6	3.6	2.7	2.4	4.6

	sc11	sc12	sc13	sc14	sc15	sc16	sc17	sc18	sc19	sc20	ave
1	9.7	10.0	8.4	8.6	7.4	7.6	6.2	6.4	5.1	5.0	7.4
2	9.4	9.7	8.0	8.2	6.8	7.0	5.3	5.5	4.2	4.1	6.8
3	9.3	9.6	7.8	8.0	6.4	6.7	5.0	5.2	3.6	3.6	6.5
v	9.4	9.8	8.1	8.3	6.9	7.1	5.5	5.8	4.3	4.2	6.9

Table S19: Performance of the seven competing methods in PCS, MTD%, above-MTD% and number of DLTs averaged over 200 random scenarios, where the MTD is randomly assigned at all K doses.

	PCS (%)	MTD%	above-MTD%	# of DLTs
			$K = 5$	
3+3	28.6	32.4	18.2	2.6
mTPI[1]	48.5	41.3	17.1	7.6
mTPI-2[1]	47.7	40.7	17.9	7.6
Keyboard[2]	52.4	38.0	20.9	–
CRM[1]	53.3	41.7	18.8	7.7
CRM[2]	53.5	42.2	21.2	–
BOIN[1]	53.0	40.8	17.7	7.6
BOIN[2]	52.2	38.1	20.5	–
BSA (fixed)	51.3	39.7	10.6	7.1
BSA (exact, early)	60.8	49.6	13.9	7.9
BSA (exact, middle)	63.6	48.0	8.4	7.3
BSA (exact, late)	53.5	40.8	8.7	7.0
			$K = 6$	
3+3	26.7	28.9	16.8	2.7
mTPI[1]	45.1	36.5	15.8	7.2
mTPI-2[1]	44.8	36.1	16.6	7.2
Keyboard[2]	49.4	34.0	19.3	–
CRM[1]	49.2	36.5	18.5	7.4
CRM[2]	50.5	37.4	20.6	–
BOIN[1]	49.7	36.1	16.5	7.2
BOIN[2]	49.2	34.0	18.9	–
BSA (fixed)	45.4	34.3	11.2	6.8
BSA (exact, early)	59.9	45.8	14.1	7.6
BSA (exact, middle)	61.7	44.0	7.9	7.0
BSA (exact, late)	49.4	35.7	8.4	6.6

Table S20: Performance of hBSA and iBOIN in PCS, MTD%, above-MTD% and number of DLTs averaged over 200 random scenarios with historical information, where the MTD is randomly assigned at all K doses.

	method	PCS (%)	MTD%	above-MTD%	# of DLTs
			correctly specified skeleton		
$K = 5$	iBOIN	66.3	43.5	16.2	–
	hBSA (fixed)	59.9	41.5	5.8	6.8
	hBSA (exact, early)	69.2	57.2	10.8	7.9
	hBSA (exact, middle)	72.5	52.4	3.7	7.1
	hBSA (exact, late)	61.9	37.8	2.4	6.3
$K = 6$	iBOIN	54.6	36.1	15.2	–
	hBSA (fixed)	57.8	41.9	4.5	6.8
	hBSA (exact, early)	65.5	51.9	11.5	7.7
	hBSA (exact, middle)	69.4	49.9	2.8	7.0
	hBSA (exact, late)	59.5	37.3	1.4	6.3
			mis-specified skeleton		
$K = 5$	iBOIN	51.7	33.7	19.8	–
	hBSA (fixed)	49.5	33.5	11.7	6.9
	hBSA (exact, early)	55.3	41.6	16.8	7.8
	hBSA (exact, middle)	61.3	41.8	9.0	7.0
	hBSA (exact, late)	49.8	29.4	9.7	6.5
$K = 6$	iBOIN	44.8	31.2	17.3	–
	hBSA (fixed)	44.0	30.3	13.5	7.0
	hBSA (exact, early)	58.3	43.9	15.8	7.7
	hBSA (exact, middle)	56.6	38.3	9.2	7.0
	hBSA (exact, late)	48.6	27.9	8.5	6.4

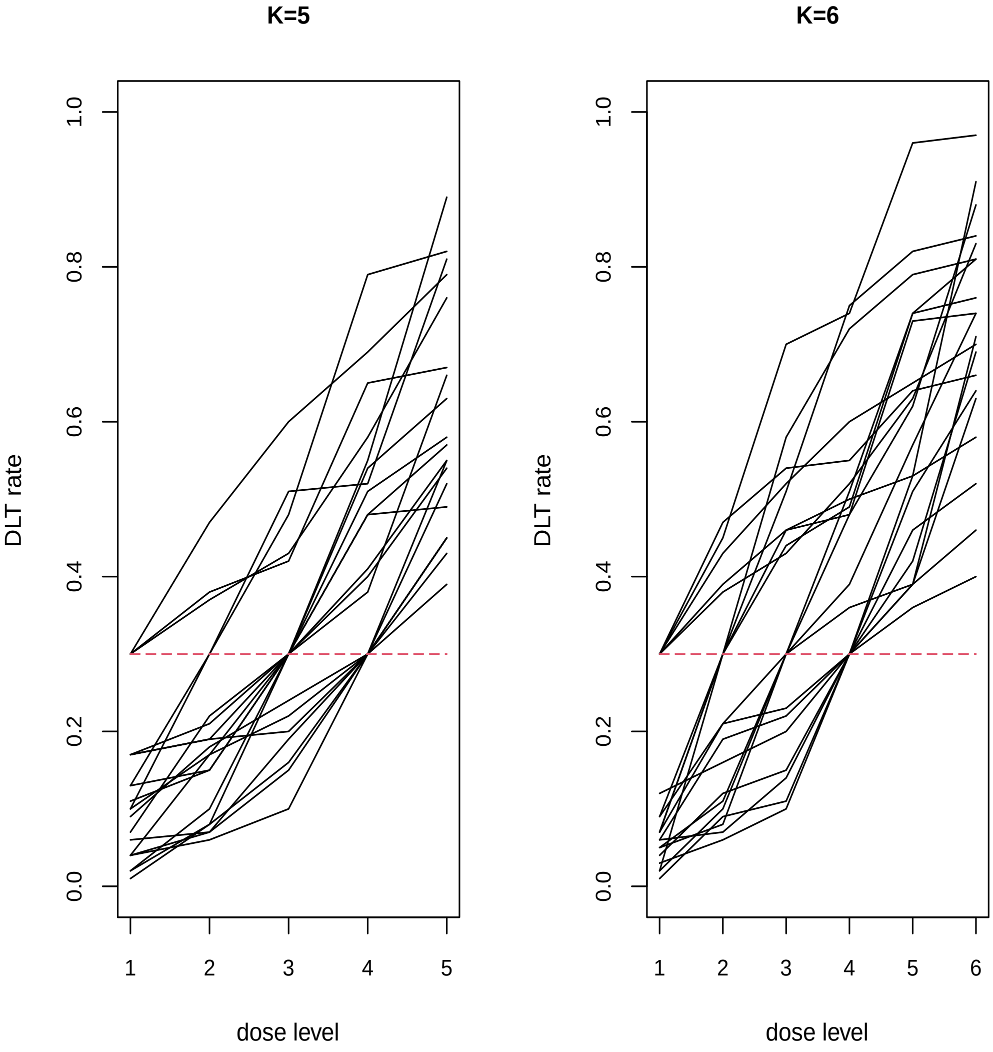

Figure S4: Twenty randomly selected scenarios of toxicity rates from the 200 scenarios generated for $K = 5$ and 6, respectively, where the target toxicity rate 30% is indicated by a horizontal dotted line.

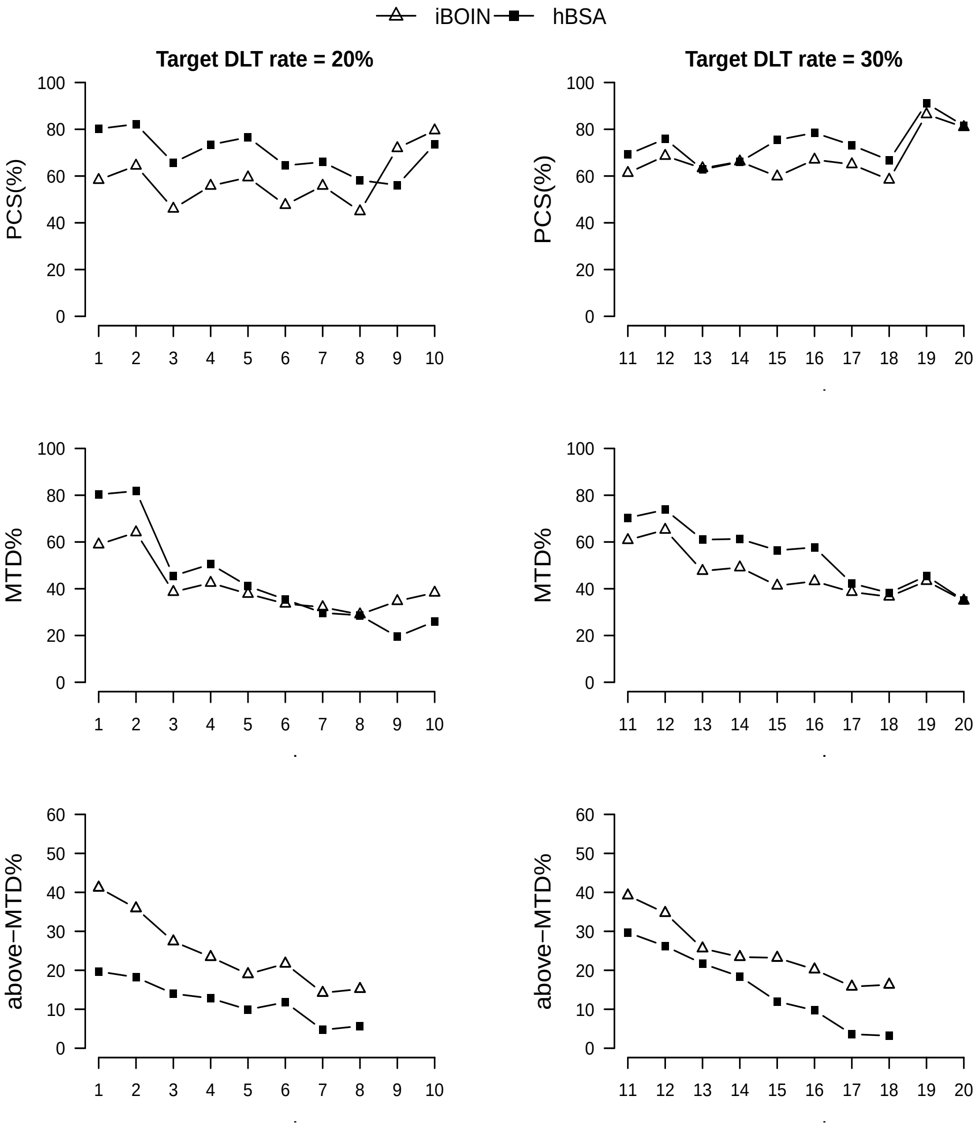

Figure S5: Performance of hBSA and iBOIN under correctly specified skeleton

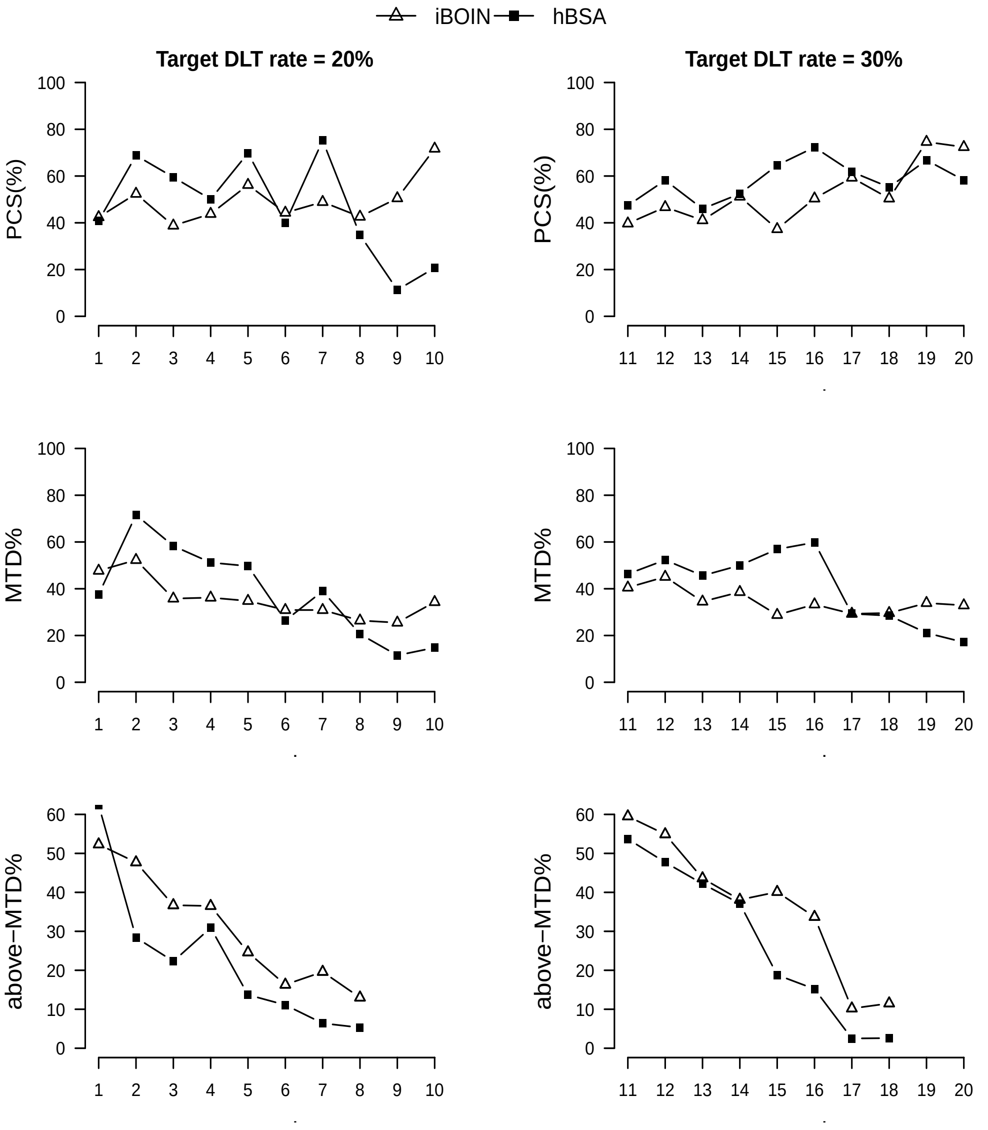

Figure S6: Performance of hBSA and iBOIN under mis-specified skeleton

References

[1] Cheung YK. Coherence principles in dose-finding studies. Biometrika. 2005;92:863–873.

[2] Yan F, Mandrekar SJ, Yuan Y. Keyboard: a novel Bayesian toxicity probability interval design for phase I clinical trials. Clinical Cancer Research. 2017;23:3994–4003.

[3] Esterni B, Mane B. UBCRM: Functions to Simulate and Conduct Dose-Escalation Phase I Studies; 2015. R package version 1.0.1. Available from: https://cran.r-project.org/package=UBCRM.

[4] Zhou Y, Lee JJ, Wang S, Bailey S, Yuan Y. Incorporating historical information to improve phase I clinical trial designs. Pharmaceutical Statistics. 2021;20:1017–1034.
